# TinkerHap—a novel read-based phasing algorithm with integrated multimethod support for enhanced accuracy

**DOI:** 10.1093/gigascience/giaf138

**Published:** 2025-10-28

**Authors:** Uri Hartmann, Eran Shaham, Dafna Nathan, Ilana Blech, Danny Zeevi

**Affiliations:** Department of Biotechnology, Jerusalem Multidisciplinary College, Jerusalem 9101001, Israel; Department of Biotechnology, Jerusalem Multidisciplinary College, Jerusalem 9101001, Israel; Department of Biotechnology, Jerusalem Multidisciplinary College, Jerusalem 9101001, Israel; Department of Biotechnology, Jerusalem Multidisciplinary College, Jerusalem 9101001, Israel; Department of Biotechnology, Jerusalem Multidisciplinary College, Jerusalem 9101001, Israel

**Keywords:** phasing, genome phasing, haplotype reconstruction, hybrid algorithm, long-read sequencing, rare variants, read-based phasing, TinkerHap, variant analysis

## Abstract

**Background:**

Phasing, the assignment of alleles to their respective parental chromosomes, is fundamental to studying genetic variation and identifying disease-causing variants. Traditional approaches, including statistical, pedigree-based, and read-based phasing, face challenges such as limited accuracy for rare variants and reliance on external reference panels.

**Results:**

To address these limitations, we developed TinkerHap, a novel phasing algorithm that integrates a read-based phaser, based on a pairwise distance-based unsupervised classification, with external phased data, such as statistical or pedigree phasing. We evaluated TinkerHap’s performance against other phasing algorithms using 1,040 parent–offspring trios from the UK Biobank (Illumina short reads) and GIAB Ashkenazi trio (PacBio long reads). TinkerHap’s read-based phaser alone achieved higher phasing accuracies than all other algorithms with 95.1% for short reads (second best: 94.8%) and 97.5% for long reads (second best: 95.5%). Its hybrid approach further enhanced short-read performance to 96.3% accuracy and was able to phase 99.5% of all heterozygous sites. TinkerHap also extended haplotype block sizes to a median of 79,449 bp for long reads (second best: 68,303 bp) and demonstrated higher accuracy for both single-nucleotide polymorphisms and indels.

**Conclusions:**

The combination of a robust read-based algorithm and a hybrid integration strategy makes TinkerHap a powerful and versatile tool for genomic analysis, enabling more accurate, contiguous, and comprehensive phasing across diverse sequencing platforms and variant types.

## Introduction

Phasing is the process of assigning alleles to their respective maternal or paternal chromosomes. It is essential for determining precise protein sequences in an individual and identifying genes that cause diseases.

Various methods of phasing are available, including statistical phasing based on phased reference genomes (e.g., ShapeIT5 [1] and Beagle [2]), pedigree-based phasing (e.g., LINKPHASE3 [3] and TrioPhaser [4]), and read-based phasing (e.g., WhatsHap [5] and HapCUT2 [6]). Statistical phasing is limited by how well the reference panel represents the sample data and is particularly inaccurate in phasing rare variants [7]. Pedigree-based phasing is accurate for common and rare variants, but pedigree information is typically not available for the inspected individual.

Read-based (or read-aware) phasing works by analyzing sequencing reads that span multiple heterozygous sites to phase them together, resulting in very high accuracy. This method is independent of statistical bias, is unaffected by the rarity of the alleles, and does not require pedigree data, although mapping bias from reference-based alignment remains a known limitation. However, since read-based phasing cannot phase regions where heterozygous sites are farther than read sizes, it can only effectively be used in highly variable regions or when using long-reads that span several heterozygous sites.

Here we present TinkerHap, a read-based phasing tool offering consistent performance and enhanced accuracy. TinkerHap excels in accurately handling rare variants and variable genomic regions, such as the human leukocyte antigen (HLA) locus, while also effectively phasing long-read data. Moreover, TinkerHap uniquely merges external phasing blocks into its read-based framework while maintaining block continuity, which differs from existing tools that do not integrate merging within a read-aware propagation step. This hybrid approach bridges gaps in read coverage and extends haplotype blocks.

## Methods

### Overview

TinkerHap is implemented in Python 3 and utilizes the *pysam* package [8] for manipulating alignment and variant calling files. The command-line interface accepts an alignment file (SAM/BAM/CRAM) and a variant call file (as variant call format (VCF) or a binary call format (BCF)) as inputs, producing a phased VCF file through read-based haplotype phasing. Optionally, TinkerHap can integrate a prephased VCF file from a third-party tool (e.g., ShapeIT5 [9] for statistical-based phasing) to align and merge haplotypes with greater accuracy when possible.

Additionally, TinkerHap can generate multiple output formats to represent the phased haplotypes: a BED file listing the identified haplotype blocks, a BAM file identical to the original but annotated with haplotype and phase information in the haplotype phase (HP) field and haplotype number (HT) field, and 2 separate BAM files—each containing reads corresponding to one of the phased alleles. These outputs facilitate annotation or the splitting of the original alignment into distinct files for each allele, enabling downstream analyses.

### Algorithm

TinkerHap implements a 3-step phasing algorithm, based on a pairwise distance-based unsupervised classification, designed for precision. Below is a detailed description of each step with mathematical notations.

#### Identification of heterozygous sites

Let $S = \{ {{s}_1,{s}_2,\ \cdots \ ,{s}_m} \}\ $ represent the set of heterozygous sites identified from the input VCF. A site ${s}_i$ is considered heterozygous if ${a}_i\, \neq \,{b}_i\ $, where ${a}_i$ and ${b}_i$ are the 2 alleles at ${s}_i$. The loci of these sites are identified as $L( S ) = \{ {{l}_1,{l}_2,\, \cdots \,,{l}_m} \}$, forming the foundation for subsequent phasing steps. Ambiguous allele calls, characterized by low scores in the VCF’s QUAL column and typically caused by sequencing or alignment errors, are flagged.

#### Association of reads with heterozygous sites

Let $R = \{ {{r}_1,{r}_2,\, \cdots \,,{r}_n} \}$ denote the set of sequencing reads. Each read ${r}_j$ spans a subset of heterozygous sites ${S}_j \subseteq S$. For each read, we map its alleles to the overlapping sites:


\begin{eqnarray*}
A\left( {{r}_j,{s}_i} \right) = \left\{ {\subseteq \begin{array}{@{}*{2}{c}@{}} {{a}_i,}&{{\mathrm{if\ allele\ matches\ allele\ 1}}}\\ {{b}_i,}&{{\mathrm{if\ allele\ matches\ allele\ 2}}} \end{array}} \right.
\end{eqnarray*}

This step ensures precise allele identification by linking reads to heterozygous sites while accounting for potential alignment errors or ambiguities, such as insertions/deletions (indels). To ensure accuracy, only reads meeting a minimum mapping quality (MAPQ) threshold (e.g., MAPQ ≥20) are considered, ensuring that low-confidence alignments do not influence the phasing process.

#### Calculation of phase scores

The phasing process begins by arbitrarily assigning the first read ${r}_1$ to one of the haplotypes, for instance, ${H}_1$. This initial assignment acts as a seed to propagate haplotypes across all overlapping reads.

Each read *r* is then evaluated to determine its phase-matching scores ${P}_1( r )$ and ${P}_2( r )$ for the 2 haplotypes, ${H}_1$ and ${H}_2$. These scores are computed by analyzing all overlapping reads and the heterozygous sites they share with *r*.

The phase scores are calculated as


\begin{eqnarray*}
{P}_H\left( r \right) = \mathop \sum \limits_{k\, \in \,{K}_r} \mathop \sum \limits_{s\, \in \,{S}_r \cap {S}_k} {\mathrm{\Delta }}{P}_H\left( r \right)
\end{eqnarray*}

where ${K}_r$ is the set of all overlapping reads for *r*, and ${S}_r$ and ${S}_k$ are the sets of heterozygous sites for reads *r* and *k*, respectively. The contribution of each shared site *s* to the phase score, ${\mathrm{\Delta }}{P}_H( r )$, is defined as


\begin{eqnarray*}
{\mathrm{\Delta }}{P}_H\left( r \right) = \left\{ {\begin{array}{@{}*{1}{c}@{}} { + w\left( s \right),{\mathrm{if\ }}{A}_r\left( s \right) = {A}_k\left( s \right){\mathrm{\ and\ }}k{\mathrm{\ belongs\ to\ haplotype\ }}H}\\ { - w\left( s \right),{\mathrm{if\ }}{A}_r\left( s \right)\, \neq \,{A}_k\left( s \right){\mathrm{\ and\ }}k{\mathrm{\ belongs\ to\ haplotype\ }}H} \end{array}} \right.
\end{eqnarray*}

Here, ${A}_r( s )$ and ${A}_k( s )$ represent the alleles of *r* and *k* at site *s*, respectively. The weight $w( s )$ assigned to site *s* depends on the type of heterozygous site:


\begin{eqnarray*}
w\left( s \right) = \left\{ {\begin{array}{@{}*{1}{c}@{}} {2,{\mathrm{if\ }}s{\mathrm{\ is\ a\ SNP}}}\\ {1,{\mathrm{if\ }}s{\mathrm{\ is\ an\ indel}}} \end{array}} \right.
\end{eqnarray*}

This scoring ensures that the phase scores for *r* are influenced by the agreement or disagreement between *r* and all overlapping reads at shared heterozygous sites.

After calculating the phase scores, the haplotype $HP$ of *r* is assigned as follows:


\begin{eqnarray*}
{\mathrm{HP}}\left( r \right) = \left\{ {\begin{array}{@{}*{1}{c}@{}} {{H}_1,{\mathrm{if\ }}{P}_1\left( r \right) > {P}_2\left( r \right),}\\ {{H}_2,{\mathrm{if\ }}{P}_2\left( r \right) > {P}_1\left( r \right),} \end{array}} \right.
\end{eqnarray*}

If ${P}_1( r )$ and ${P}_2( r )$ are equal, the haplotype assignment can propagate from the overlapping read with the strongest phase connection, or a new haplotype block may be started. This approach ensures consistency in haplotype assignments based on the majority consensus among overlapping reads.

#### Haplotype extension

Haplotypes are extended iteratively by analyzing overlapping reads. If a read ${r}_k$ overlaps 2 or more phased reads $\{ {{r}_{{j}_1},{r}_{{j}_2},\, \cdots \,} \}$, its phase is determined by propagating the majority consensus:


\begin{eqnarray*}
{\mathrm{HP}}\left( {{r}_k} \right) = \left\{ {{\mathrm{HP}}\left( {{r}_{{j}_1}} \right),{\mathrm{\ HP}}\left( {{r}_{{j}_2}} \right),\, \cdots \,} \right\}
\end{eqnarray*}

This ensures the consistency of haplotype assignments across contiguous genomic regions. Reads that span conflicting haplotypes are flagged for manual review or downstream quality filtering.

#### Pair-end read merging

For paired-end reads $( {{r}_i,{r}_j} )$, the algorithm evaluates the consistency of their haplotypes:


\begin{eqnarray*}
M\left( {{r}_i,{r}_j} \right) = \left\{ {\begin{array}{@{}*{2}{c}@{}} { + 1,}&{{\mathrm{if\ HP}}\left( {{r}_i} \right) = {\mathrm{HP}}\left( {{r}_j} \right)}\\ { - 1,}&{{\mathrm{if\ HP}}\left( {{r}_i} \right)\, \neq \,{\mathrm{HP}}\left( {{r}_j} \right)} \end{array}} \right.
\end{eqnarray*}

Inconsistent pairs trigger a phase reassignment to minimize discordance, leveraging the paired-end linkage information. A weighted graph representation of pair-end links can be constructed for further optimization of haplotype continuity.

#### Integration with prephased data (optional)

When an additional prephased VCF file is provided (e.g., one generated by statistical phasing tools such as ShapeIT5), the algorithm merges the read-based haplotypes with the prephased data. This involves merging haplotypes and, if necessary, switching the phase numbers (e.g., swapping haplotype 1 and haplotype 2) to ensure consistency with the prephased data numbering of haplotypes.

The alignment score $A( {b,h} )$ for a prephased block *b* and a read-based block *h* is calculated as


\begin{eqnarray*}
A\left( {b,h} \right) = \mathop \sum \limits_{s\, \in \,{S}_b \cap {S}_h} w\left( s \right)
\end{eqnarray*}

where ${S}_b$ and ${S}_h$ are the sets of heterozygous sites in *b* and *h*, and $w( s )$ represents the weight based on the site type (e.g., single-nucleotide polymorphisms [SNP] or indel). Haplotypes are adjusted to maximize $A( {b,h} )$, ensuring that the merged haplotypes align with the prephased data and improving the overall phasing accuracy.

#### Output generation

The final outputs include the following:

1. Phased VCF: Annotated with a phase set (PS) field.
2. Annotated BAM: Each read is tagged with HP and HT fields.
3. Split BAM files: Separate BAM files for each haplotype, facilitating downstream analyses.
4. BED file: Haplotype boundaries across the genome are defined for visualization.

The detailed algorithm and code can be accessed at GitHub (see Availability of Source Code and Requirements).

Supplementary material for this article, including detailed descriptions of the evaluation and benchmarking procedures, all methodological steps, permissible data, scripts, and resources necessary for reproducibility, is available at Github (see Availability of Supporting Source Code and Requirements).

### Evaluation

TinkerHap was evaluated in the following use cases:

1. To evaluate the algorithm’s performance in variable regions and for rare variants using Illumina short reads, we analyzed whole-genome sequencing (WGS) data from 1,040 parent–offspring trios that we identified in the UK Biobank [10] on the major histocompatibility complex (MHC) class II region in humans, specifically on chr6:32,439,878–33,143,325 (hg38 genome version).
2. To evaluate the algorithm’s performance with long reads, we used PacBio long-read sequencing data of the full genomes of Genome in a Bottle (GIAB) Ashkenazi trio HG002-4 and Chinese trio HG005-7 datasets by Revio (publicly offered by GIAB [11]). Results were averaged across the Ashkenazi and Chinese GIAB trios.

For each offspring in the trios, we constructed a “truth” set of known phased heterozygous sites (“truth sites”). This was achieved by examining loci where each parent possesses different homozygous alleles or where 1 parent was heterozygous, and the other was homozygous. After preparing the data, we phased the offspring sequence using the following algorithms: ShapeIT5 [9], WhatsHap [5], HapCUT2 [6], TinkerHap, and TinkerHap with ShapeIT5 [9] phased data as an additional input for merging haplotypes (as described in the “Algorithm” section above). The success rate was evaluated by counting the number of sites in the phased output that matched the truth set.

All algorithms were run on the same virtual instance type—UK BioBank instance type “mem3_ssd1_v2_x2” (2 cores, 16 GB memory, 75 GB storage).

## Results

### MHC class II gene region phasing (chr6:32,439,878–33,143,325)

**Figure 1:**
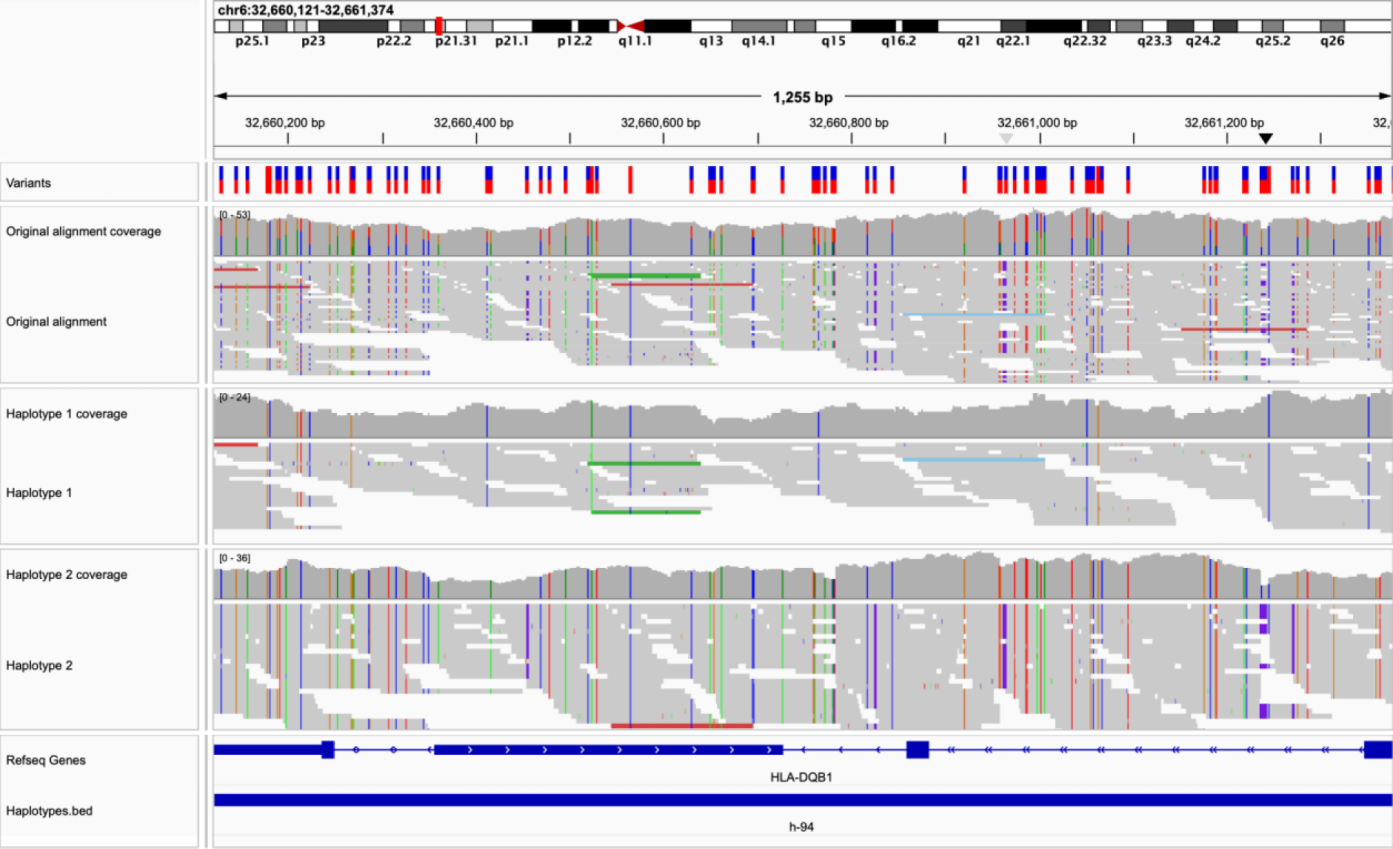
Phased BAM outputs displaying heterozygous sites (IGV [12] screenshot). The upper track is the original alignment, while the 2 tracks below represent the output of haplotype 1 and haplotype 2. Heterozygous sites are correctly segregated between the 2 phases, demonstrating successful phasing. The continuous blue line in the bottom track illustrates a BED file annotation, highlighting the size of the haplotype region where all variants are confirmed to share the same phase.

In order to assess TinkerHap's performance, we benchmarked it both in standalone mode and in hybrid mode with ShapeIT5 integration, comparing it against other leading phasing algorithms on short-read sequencing data from the highly polymorphic MHC class II region. The evaluation included metrics such as phasing accuracy, haplotype block length, and computational efficiency, as summarized in Table 1. Figure 1 illustrates the corresponding phased alignments, showing the clear segregation of heterozygous sites across haplotypes and confirming the consistency of the phasing results.

**Table 1:** Phasing performance of different algorithms on short reads aligned to the MHC class II region

Criteria	TinkerHap+ ShapeIT5^1^	TinkerHap	WhatsHap	HapCUT2	ShapeIT5
Phased %	99.5%	97.1%	86.5%	96.2%	70.5%
Phasing accuracy %^2^	96.3%	95.1%	84.9%	94.8%	70.2%
Phasing accuracy % (SNPs only)	97.1%	96.0%	86.0%	95.8%	71.6%
Phasing accuracy % (indels only)	89.6%	87.8%	76.9%	87.4%	59.6%
Haplotype size (bp)^3^	21,813	631	75	644	702,123
Phase blocks^4^	11.5	82	325	84	1
Phase blocks N50^5^ (bp)	139,281	19,596	1,489	13,678	702,123
Total phased size (bp)	623,829	329,037	119,554	276,724	702,123
Common phased site errors^6^	0.14%	0.14%	0.18%	0.12%	0.17%
Coverage	×32.5				
Heterozygous sites^7^	5,086				
Runtime (s)^8^	6.8	6.5	23.8	9.2	13.7
Bases/second	104,005	108,245	29,532	76,105	51,545
Heterozygous sites/second	743	773	211	544	368
Memory usage (MB)	109	106	397	594	24

1 TinkerHap + ShapeIT5: TinkerHap algorithm when used with additional ShapeIT5 prephased file.

2 Phasing accuracy: Successfully phased sites divided by the total number of heterozygous sites.

3 Haplotype size: Median haplotype size across all samples. “Haplotype” refers to a set of alleles at variant sites along a single chromosome that are inherited together and are guaranteed to be phased together by the algorithm.

4 Phase blocks: Median number of phase blocks across all samples.

5 Phase blocks N50: median of the N50 haplotype block sizes computed individually for each sample, providing a summary measure of typical haplotype block contiguity across the dataset.

6 Common phased sites errors: Phasing error % in heterozygous sites phased by all algorithms.

7 Heterozygous sites: Median number of heterozygous sites across all samples.

8 Runtime: Median runtime per sample.

### PacBio phasing (whole genome)

To further evaluate TinkerHap's performance, we tested it on long-read whole-genome sequencing data from the GIAB Ashkenazi and Chinese trios using PacBio technology. Similar to the short-read experiments, TinkerHap was assessed both in standalone mode and in hybrid mode with ShapeIT5 integration, and its results were compared against other leading phasing algorithms. Table 2 summarizes the performance across key metrics, including phasing accuracy, haplotype block size, and computational efficiency..

**Table 2:** Phasing performance of different algorithms on long reads of full genomes

Criteria	TinkerHap + ShapeIT5	TinkerHap	WhatsHap	HapCUT2	ShapeIT5
Phased %	99.8%	99.4%	96.8%	96.7%	87.84%
Phasing accuracy %^1^	97.8%	97.5%	95.5%	95.4%	86.68%
Phasing accuracy % (SNPs only)	98.0%	97.8%	96.5%	96.4%	89.0%
Phasing accuracy % (indels only)	96.35%	96.0%	90.2%	90.5%	75.25%
Haplotype size (bp)^2^	809,769	79,449	68,303	72,220	133,491,606
Phase blocks^3^	1,490	10,061	10,561	10,586	22
Phase blocks N50^4^ (bp)	4,603,981	614,204	558,942	558,942	133,491,606
Total phased size (Mbp)	2,675	2,275	2,173	2,171	2,807
Common phased site errors^5^	0.14%	0.14%	0.15%	0.20%	0.60%
Coverage	×50				
Heterozygous sites	100,910				
Runtime (s)^6^	10,797	10,519	17,578	5,495	27,474
Bases/second	290,572	298,239	178,475	570,891	114,191
Heterozygous sites/second	421	433	259	829	166
Memory usage (MB)	1,780	1,726	1,696	876	2,189

1 Phasing accuracy: Successfully phased sites divided by the total number of heterozygous sites.

2 Haplotype size: Median haplotype size across all samples.

3 Phase blocks: Average number of phase blocks across all samples.

4 Phase blocks N50: median of the N50 haplotype block sizes computed.

5 Common phased sites errors: Phasing error % in heterozygous sites phased by all algorithms.

6 Runtime: Average runtime per sample.

## Discussion

Here, we introduce TinkerHap, a read-based phasing algorithm designed for accurate and reliable phasing across diverse genomic contexts, with the ability to integrate statistical phasing data from third-party tools for improved performance.

We evaluated TinkerHap using 2 datasets: (i) the MHC class II region in humans with Illumina short-read WGS data to assess its accuracy in variable regions and (ii) PacBio sequencing data to evaluate its performance with long reads. These datasets were selected due to their suitability for testing read-based phasing algorithms, as both are characterized by a high density of variants that provide many opportunities for phasing.

### Performance of short reads in variable regions

In the MHC class II region using short reads, TinkerHap phased 97.1% of variants with 95.1% accuracy. In comparison, the second-best algorithm phased 96.2% of variants with 94.8% accuracy. All methods showed higher phasing accuracy for SNPs compared to indels (97.1% and 89.6%, respectively, in TinkerHap).

### Performance of long-read sequencing

TinkerHap achieved a phasing accuracy of 97.5% for SNPs and 96.0% for indels with PacBio datasets. These results were superior to the second-best algorithm, which demonstrated accuracies of 95.5% and 95.4%, respectively. Moreover, TinkerHap produced longer haplotype blocks (median size: 79,449 bp) compared to the second-best algorithm (68,303 bp). Runtime analysis revealed that TinkerHap required 10,519 seconds per sample, compared to 5,495 seconds for the fastest algorithm.

### Comparison of long-read and short-read performance

An important advantage of TinkerHap, as well as all read-based algorithms, is that it is less likely to be affected by the rarity of a variant [7]. However, the density of the variants and the length of the sequencing read are key factors for the performance of TinkerHap. A key limitation of read-based phasing approaches, including TinkerHap, arises from the typical length of short sequencing reads (~150 bp) relative to the spacing between variants (500–1,000 bp on average). As a result, individual reads seldom span multiple variants, thereby yielding little to no additional or informative phasing data. This limitation confines the effective application of read-based phasing methods like TinkerHap primarily to long-read sequencing projects, where reads are sufficiently extended to span multiple variants, or to genomic regions exhibiting high variant density (greater than approximately 1 variant per 100 bp), such as the HLA locus.

Accordingly, TinkerHap performed better with long-read sequencing data compared to short-read data in several key metrics. Long reads offer superior upstream alignment quality, particularly at highly variable sites, which enhances the overall accuracy of variant calling and subsequent phasing steps. Long-read data yielded more extensive haplotype blocks (median size: 79,449 bp compared to 631 bp with short reads) and higher phasing accuracy (97.5% for SNPs in long reads compared to 96.0% in short reads, as well as 96% for indels in long reads compared to 87.8% in short reads). This improved performance is expected due to long reads containing more heterozygote sites and enabling improved alignments.

### Integration with statistical phasing

TinkerHap uniquely includes the ability to integrate data from third-party tools, such as ShapeIT5. By incorporating prephased haplotypes, the TinkerHap + ShapeIT5 combination achieved 99.5% phased variants with 96.3% accuracy, significantly outperforming standalone methods. This hybrid approach improved haplotype block continuity and effectively addressed gaps in read coverage.

### Limitations

TinkerHap’s runtime and memory usage for long-read data present areas for potential optimization, and it currently lacks support for polyploid genomes. TinkerHap is limited in merging distant haplotypes, which could be particularly useful for applications such as exome sequencing. Future incorporation of pedigree information could address this issue and enhance TinkerHap’s accuracy in trio or family-based studies.

In most phasing errors that we manually examined, inaccuracies were primarily attributed to upstream variant calling rather than to the phasing algorithm itself. This suggests that TinkerHap may be approaching the limit of what can be achieved with downstream read-based phasing alone. This underscores the importance of high-quality preprocessing.

## Supplementary Material

giaf138_Supplemental_File

giaf138_Authors_Response_To_Reviewer_Comments_Original_Submission

giaf138_GIGA-D-25-00150_Original_Submission

giaf138_GIGA-D-25-00150_Revision_1

giaf138_Reviewer_1_Report_Original_Submission Arang Rhie -- 6/9/2025

giaf138_Reviewer_2_Report_Original_Submission Yilei Fu, Ph.D. -- 6/15/2025

giaf138_Reviewer_2_Report_Revision_1 Yilei Fu, Ph.D. -- 10/3/2025

giaf138_Reviewer_3_Report_Original_Submission Julia Markowski -- 6/25/2025

## Data Availability

**UK Biobank data:** The Illumina short-read data from 1,040 parent–offspring trios used in this study were accessed from the UK Biobank under application number 74655. These data are available under controlled access due to participant privacy considerations. Researchers can apply for access through the UK Biobank Access Management System by submitting a detailed research proposal. Further information and application guidelines are available at [13]. **Genome in a Bottle (GIAB) data:** The long-read sequencing data from the GIAB Ashkenazi and Chinese trios used for algorithm evaluation are publicly available at the National Center for Biotechnology Information (NCBI) FTP site. The datasets can be accessed directly via ftp [14]. Further information is available in the supplementary material for this article. All supporting data are available in *GigaScience* repository, GigaDB [15].

## Availability of Supporting Source Code and Requirements

Project name: TinkerHap

Project homepage: https://github.com/DZeevi-Lab/TinkerHap

Supplementary information: https://github.com/DZeevi-Lab/TinkerHap-Supplementary

Operating system(s): Platform independent

Programming language: Python

Other requirements: Python 3.6.0 or higher, pysam 0.17 or higher

License: MIT

RRID:SCR_027235

bio.tools ID: tinkerhap

## Abbreviations

BCF: binary call format; GIAB: Genome in a bottle; HLA: human leukocyte antigen; HP: haplotype phase; HT: haplotype number; indels: insertions/deletions; MAPQ: mapping quality; MHC: major histocompatibility complex; PS: phase set; SNP: single-nucleotide polymorphism; VCF: variant call file; WGS: whole-genome sequencing.
