# Supplementary material for: TinkerHap—a novel read-based phasing algorithm with integrated multimethod support for enhanced accuracy
Source: Gigascience. 2025 Oct 28;14:giaf138. doi: 10.1093/gigascience/giaf138 (PMC12723663; doi:10.1093/gigascience/giaf138)
Supplement: giaf138_Supplemental_File [file giaf138_supplemental_file.docx]

**Supplementary information for TinkerHap - A Novel Read-Based Phasing Algorithm with Integrated Multi-Method Support for Enhanced Accuracy**

Uri Hartmann^1*^, Eran Shaham^1^, Dafna Nathan^1^, Ilana Blech^1^, Danny Zeevi^1^

^1^Department of Biotechnology, Jerusalem Multidisciplinary College, Jerusalem, Israel.

*Corresponding author. Email: uri.hartman@edu.jmc.ac.il

# **1. Overview**

This document details the evaluation and benchmarking procedures used in the study, including all steps, data (where permissible), scripts, and resources required for reproducibility. All scripts referenced in this document are available at <https://github.com/DZeevi-Lab/TinkerHap-Supplementary>

# **2. Tools used**

The tools referenced throughout this document are listed below:

| **Tool** | **Version** | **Availability** |
| --- | --- | --- |
| **ShapeIT** | 5.1.1 | [https://github.com/odelaneau/shapeit5](https://github.com/odelaneau/shapeit5/) |
| **WhatsHap** | 2.3 | <https://github.com/whatshap/whatshap> |
| **HapCUT2** | 1.3.4 | <https://github.com/vibansal/HapCUT2> |
| **pysam** | 0.22.1 | <https://github.com/pysam-developers/pysam> |
| **samtools** | 1.21 | <https://github.com/samtools/samtools> |
| **bcftools** | 1.21 | <https://github.com/samtools/bcftools> |

# **3. MHC class II gene region phasing evaluation**

## For the MHC class II gene region phasing evaluation we analyzed Whole Genome Sequencing (WGS) data from 1,040 parent-offspring trios that we identified in the UK Biobank on the MHC class II region in humans, specifically on chr6:32,439,878-33,143,325 (hg38 genome version)

## 3.1. Identifying trios

Trios were identified using three input files:

1. ukb_rel.dat – Kinship file fetched from the UK BioBank Bulk folder. Its contents are described at <https://biobank.ndph.ox.ac.uk/ukb/ukb/docs/ukb_genetic_data_description.txt>
2. ukbiobank_agesex.csv – Age and sex file for each of the samples in the UK BioBank (fields are: eid, p22001, p34)
3. all-ids.csv – A list of all the sample IDs in the UK BioBank

The script **01-families-lookup.py** was then used to generate mother-father-offspring trios. The script identifies the trios by using the criteria: 0.177 ≤ kinship ≤ 0.354 and ibs0 < 0.0012 to establish a parent-offspring relationship (as suggested at <https://www.kingrelatedness.com/manual.shtml>), and the sex + age gap to determine whether the relationship is mother-offspring or father-offspring.

The script outputs two files:

1. uk-trios.txt - Tab Delimited file of the trios found: Mother, Father, Offspring, Number of siblings, and Years of Birth of the trio samples.
2. uk-offsprings-samples.txt - Bash array format of the offsprings and parents to be used in bash scripts.

## 3.2. Extracting “truth” dataset from trios

A truth dataset for evaluating the algorithms was extracted from the trios using the **02-build-truth.py** script. The script accepts the “uk-offsprings-samples.txt” file created at the previous step, a path where the input VCF files can be found and an output path. For each trio, the script creates a pickle (pkl) file containing a dictionary of the offspring’s heterozygous variants and, wherever possible, their expected phased genotypes based on the parents’ information in variants where at least one of the parents is homozygote.

## 3.3. Phasing

Four phasing algorithms were used for evaluation using each offspring’s VCF/CRAM files. As a preliminary step we used bcftools and samtools to create an input file for each offspring for the selected region only (samtools/bcftools view <offspring> chr6:28510120-33480577 -o <offspring>). These files were then used as inputs for the various algorithms. The command-line execution of each algorithm was evaluated for timing and memory usage using the “time -v” command (<https://man7.org/linux/man-pages/man1/time.1.html>). All algorithms were run on the same virtual instance type – UK BioBank instance type “mem3_ssd1_v2_x2” (2 cores, 16GB memory, 75GB storage).

- **ShapeIT**: We’ve used the 1kGP high-coverage reference panel from <https://ftp.1000genomes.ebi.ac.uk/vol1/ftp/data_collections/1000G_2504_high_coverage/working/20220422_3202_phased_SNV_INDEL_SV/1kGP_high_coverage_Illumina.chr6.filtered.SNV_INDEL_SV_phased_panel.vcf.gz>
  The following command line was used for each offspring:
  - phase_common_static \
     --input <OFFSPRING.vcf.gz> \
     --reference reference_panel.vcf.gz \
     --map chr6.b38.gmap.gz \
     --output shapeit-<OFFSPRING>.bcf \
     --region chr6 \
     --threads 1
- WhatsHap: The following command line was used for each offspring:
  - whatshap \
     phase \
     -o whatshap-<OFFSPRING>.vcf.gz \
     --reference= hs38DH.fa \
     <OFFSPRING.vcf.gz> \
     <OFFSPRING.cram>
- HapCUT2: The following two command lines were used for each offspring:
  - extractHAIRS \
     --bam <OFFSPRING.cram> \
     --VCF <OFFSPRING.vcf.gz> \
     --ref hs38DH.fa \
     --indels 1 \
     --out fragment1
  - HAPCUT2 \

--fragments fragment1 \

--VCF "child.vcf" \

--out hapcut2-<OFFSPRING>.vcf.gz

- TinkerHap was run twice per offspring, once as a standalone tool and once using the ShapeIT output as a reference scaffold. For the standalone run, the following command line was used for each offspring:
  - python3 tinkerhap.py \
     --vcf-in <OFFSPRING.vcf.gz> \
     --bam-in <OFFSPRING.cram> \
     --vcf-out tinkerhap-<OFFSPRING>.vcf.gz

For the TinkerHap + ShapeIT combination, the following command line was used for each offspring:

- - python3 tinkerhap.py \
     --vcf-in <OFFSPRING.vcf.gz> \
     --vcf-scaffold shapeit-<OFFSPRING>.bcf \
     --bam-in <OFFSPRING.cram> \
     --vcf-out tinkerhap-si-<OFFSPRING>.vcf.gz

## 3.4. Evaluating results

Evaluation comprised four phases: parsing timing results, assessing the quality of each offspring phasing in each algorithm, parsing common sites errors, and grouping the assessment and timing results.

- Timing and memory usage logs from the “time -v” referenced in previous step was analyzed using the **03-analyze-timings.py** script. The script outputs memory usage and execution time for each sample in each of the different algorithms used. The output of the script with full information can be found in the **mhc2short-time-mem.txt** file (note: sample identifiers have been obfuscated for privacy).
- Quality assessment of each offspring was analyzed using the **04-offspring-tester.py** script. Using the "Truth" pickle files created in an earlier step, the script was used to identify correctly phased heterozygous variants, identify phasing errors - instances where the phase between two consecutive heterozygous sites within a haplotype block is incorrect relative to the truth - quantify sites present in the truth dataset but missing or incorrectly phased in the input VCF, and generate a summary report along with detailed error logs.
  The script generates a pickle file detailing the tested variants, indicating whether each locus is an SNP or indel, and whether the phasing was successful or not. In addition, it generates a detailed output file that includes summary statistics and full information about the phasing errors and appends the summary information into a tab-delimited text file that is updated with each run. Each line summarizes a single test run, including: sample name, method name, total sites from the truth set, total SNP sites from the truth set, number of errors (overall and for SNPs), number of missing sites (overall and for SNPs), success percentage (overall and for SNPs), number of distinct haplotype blocks found in the input VCF, and number of haplotype blocks containing only a single variant. The output of the script with full information can be found in the **mhc2short-detailed.txt** file (note: sample identifiers have been obfuscated for privacy).
  *Note*: The script's command-line usage is identical across algorithms, except for ShapeIT, where haplotypes are ignored (--ignore-haplotypes) as the output always contains one large haplotype.
- The script **05-analyze-common-sites.py** analyzes phasing consistency across methods by evaluating common loci. It processes the pickle files generated in the previous quality assessment phase and identifies positions that were phased by all algorithms for each sample. For these shared loci, it computes the error rate per method, enabling a direct comparison across algorithms on a common ground. The output of the script with full information can be found in the **mhc2short-common-sites.txt** file (note: sample identifiers have been obfuscated for privacy).
- As a final step of the evaluation, the script **06-summary.py** parses the three text files generated by previous steps and outputs the summary table included in the article. The output of the script with full information can be found in the **mhc2short-summary.txt** file.

# **4. PacBio whole genome phasing evaluation**

## For the PacBio whole-genome phasing evaluation, we used PacBio long-read sequencing data of the full genomes of GIAB Ashkenazi trio HG002-4 and Chinese trio HG005-7 datasets by Revio.

## 4.1. Identifying trios

Two trios were used for the evaluation.

- GIAB “Ashkenazim” trio VCF files were retrieved from <https://ftp-trace.ncbi.nlm.nih.gov/ReferenceSamples/giab/data/AshkenazimTrio/analysis/PacBio_HiFi-Revio_20231031/pacbio-wgs-wdl_germline_20231031/> (HG002-4.GRCh38.deepvariant.g.vcf.gz files)
  Offspring BAM file was retrieved from <https://ftp-trace.ncbi.nlm.nih.gov/ReferenceSamples/giab/data/AshkenazimTrio/HG002_NA24385_son/PacBio_HiFi-Revio_20231031/HG002_PacBio-HiFi-Revio_20231031_48x_GRCh38-GIABv3.bam>
- GIAB “Chinese” trio VCF files were retrieved from <https://ftp-trace.ncbi.nlm.nih.gov/ReferenceSamples/giab/data/ChineseTrio/analysis/PacBio_CCS_15kb_20kb_chemistry2_12072020/> (HG005-7.GRCh38.deepvariant.vcf.gz)
  BAM file was retrieved from <https://ftp-trace.ncbi.nlm.nih.gov/ReferenceSamples/giab/data/ChineseTrio/HG005_NA24631_son/PacBio_CCS_15kb_20kb_chemistry2/GRCh38/GIAB_5mC_CpG/HG005.GRCh38.deepvariant.haplotagged.bam>

## 4.2. Preparing input files

## To optimize analysis and streamline truth dataset extraction of PacBio sequencing data across all phasing algorithms, the original BAM and VCF files were first split by chromosome. This strategy significantly reduced computational resource requirements, making the pipeline more manageable on standard computational infrastructure. Additionally, chromosome-level processing improved transparency and interpretability of the phasing performance by allowing method behavior to be evaluated independently across different genomic regions. Extraction was performed using the following command line: samtools/bcftools view <offspring> chr<1..22> -o <offspring>-chr<1..22>

## 4.3. Extracting “truth” dataset from trios

Truth dataset for evaluating the various algorithms was extracted from the trios using the **02-build-truth.py** script in the same manner as described above on item 3.2.

## 4.4. Phasing

Phasing commands for PacBio data were the same for ShapeIT, WhatsHap and TinkerHap as those described in section 3.3 above.

The command line for HapCUT2 was the same as section 3.3 above but also included the “--pacbio 1” option.

## 4.5. Evaluating results

Evaluating the results was done in four phases similar to item 3.4 above, except for the **05-analyze-common-sites.py** and **06-summary.py** scripts were used with “--pacbio” optional parameter. The output of these results can be found in the **pacbio-time-mem.txt**, **pacbio-detailed.txt**, **pacbio-common-sites.txt**, and **pacbio-summary.txt** files.
