# Supplementary material for: TinkerHap—a novel read-based phasing algorithm with integrated multimethod support for enhanced accuracy
Source: Gigascience. 2025 Oct 28;14:giaf138. doi: 10.1093/gigascience/giaf138 (PMC12723663; doi:10.1093/gigascience/giaf138)
Supplement: giaf138_GIGA-D-25-00150_Original_Submission [file giaf138_giga-d-25-00150_original_submission.pdf]

GigaScience

TinkerHap - A Novel Read-Based Phasing Algorithm with Integrated Multi-Method Support for Enhanced Accuracy

--Manuscript Draft--

**Manuscript Number:**	GIGA-D-25-00150
**Full Title:**	TinkerHap - A Novel Read-Based Phasing Algorithm with Integrated Multi-Method Support for Enhanced Accuracy
**Article Type:**	Technical Note
**Funding Information:**	Juvenile Diabetes Research Foundation in Israel (2658/21) — Dr. Danny Zeevi<br>Israel Science Foundation (2658/21) — Dr. Danny Zeevi
**Abstract:**	Phasing, the assignment of alleles to their respective parental chromosomes, is fundamental to studying genetic variation and identifying disease-causing variants. Traditional approaches, including statistical, pedigree-based, and read-based phasing, face challenges such as limited accuracy for rare variants, reliance on external reference panels, and constraints in regions with sparse genetic variation.<br>To address these limitations, we developed TinkerHap, a novel and unique phasing algorithm that integrates a read-based phaser, based on a pairwise distance-based unsupervised classification, with external phased data, such as statistical or pedigree phasing. We evaluated TinkerHap's performance against other phasing algorithms using 1,040 parent-offspring trios from the UK Biobank (Illumina short-reads) and GIAB Ashkenazi trio (PacBio long-reads). TinkerHap's read-based phaser alone achieved higher phasing accuracies than all other algorithms with 95.1% for short-reads (second best: 94.8%) and 97.5% for long-reads (second best: 95.5%). Its hybrid approach further enhanced short-read performance to 96.3% accuracy and was able to phase 99.5% of all heterozygous sites. TinkerHap also extended haplotype block sizes to a median of 79,449 base-pairs for long-reads (second best: 68,303 bp) and demonstrated higher accuracy for both SNPs and indels. This combination of a robust read-based algorithm and hybrid strategy makes TinkerHap a uniquely powerful tool for genomic analyses.
**Corresponding Author:**	Uri Hartmann<br>Hadassah Academic College<br>Jerusalem, ISRAEL
**Corresponding Author Secondary Information:**	
**Corresponding Author's Institution:**	Hadassah Academic College
**Corresponding Author's Secondary Institution:**	
**First Author:**	Uri Hartmann
**First Author Secondary Information:**	
**Order of Authors:**	Uri Hartmann<br>Eran Shaham<br>Dafna Nathan<br>Ilana Blech<br>Danny Zeevi
**Order of Authors Secondary Information:**	
**Additional Information:**	
**Question**	**Response**

Powered by Editorial Manager® and ProduXion Manager® from Aries Systems Corporation

Are you submitting this manuscript to a special series or article collection?	No
**Experimental design and statistics**<br><br>Full details of the experimental design and statistical methods used should be given in the Methods section, as detailed in our Minimum Standards Reporting Checklist. Information essential to interpreting the data presented should be made available in the figure legends.<br><br>Have you included all the information requested in your manuscript?	Yes
**Resources**<br><br>A description of all resources used, including antibodies, cell lines, animals and software tools, with enough information to allow them to be uniquely identified, should be included in the Methods section. Authors are strongly encouraged to cite Research Resource Identifiers (RRIDs) for antibodies, model organisms and tools, where possible.<br><br>Have you included the information requested as detailed in our Minimum Standards Reporting Checklist?	Yes
**Availability of data and materials**<br><br>All datasets and code on which the conclusions of the paper rely must be either included in your submission or deposited in publicly available repositories (where available and ethically appropriate), referencing such data using a unique identifier in the references and in the "Availability of Data and Materials" section of your manuscript.<br><br>Have you have met the above requirement as detailed in our Minimum	Yes

Standards Reporting Checklist?	
GigaScience has policies and guidelines in place for the use of generative AI-writing tools such as ChatGPT. If you have used such writing tools to assist with writing the manuscript this must be declared and cited in the text. Authors should not list AI-writing tools and other AI-assisted technologies as an author or co-author and should acknowledge that they are fully responsible for text generated or refined by AI-writing tools.<p><br><br>A summary of use (particularly in the introduction or among methods) needs to be included at the end of the paper, and the outputs should also be included as a supplementary file hosted in GigaDB or other open repositories. Please read our guidelines for more information. <p><br><br>By submitting to GigaScience, you are aware of the journal's AI-writing tools policy, and if you have declared use of such tools below, you have acknowledged this where appropriate in your manuscript and have made a summary of use and outputs available. <p><br><b>AI-assisted writing tools have been used in the preparation of this manuscript?	No

Manuscript Click here to access/download;Manuscript;TinkerHap-Article-20250521 (1).docx

TinkerHap - A Novel Read-Based Phasing Algorithm with Integrated Multi-Method Support for Enhanced Accuracy

Uri Hartmann[1*], Eran Shaham[1], Dafna Nathan[1], Ilana Blech[1], Danny Zeevi[1]

[1]Department of Biotechnology, Jerusalem Multidisciplinary College, Jerusalem, Israel.

*Corresponding author. Email: uri.hartman@edu.jmc.ac.il

Abstract

Phasing, the assignment of alleles to their respective parental chromosomes, is fundamental to studying genetic variation and identifying disease-causing variants. Traditional approaches, including statistical, pedigree-based, and read-based phasing, face challenges such as limited accuracy for rare variants, reliance on external reference panels, and constraints in regions with sparse genetic variation.

To address these limitations, we developed TinkerHap, a novel and unique phasing algorithm that integrates a read-based phaser, based on a pairwise distance-based unsupervised classification, with external phased data, such as statistical or pedigree phasing. We evaluated TinkerHap's performance against other phasing algorithms using 1,040 parent-offspring trios from the UK Biobank (Illumina short-reads) and GIAB Ashkenazi trio (PacBio long-reads). TinkerHap's read-based phaser alone achieved higher phasing accuracies than all other algorithms with 95.1% for short-reads (second best: 94.8%) and 97.5% for long-reads (second best: 95.5%). Its hybrid approach further enhanced short-read performance to 96.3% accuracy and was able to phase 99.5% of all heterozygous sites. TinkerHap also extended haplotype block sizes to a median of 79,449 base-pairs for long-reads (second best: 68,303 bp) and demonstrated higher accuracy for both SNPs and indels. This combination of a robust read-based algorithm and hybrid strategy makes TinkerHap a uniquely powerful tool for genomic analyses.

Introduction

Phasing is the process of assigning alleles to their respective maternal or paternal chromosomes. It is essential for determining precise protein sequences in an individual and identifying genes that cause diseases.

Various methods of phasing are available, including statistical phasing based on phased reference genomes (e.g. ShapeIT [1] and Beagle [2]), pedigree-based phasing (e.g. LINKPHASE3 [3] and TrioPhaser [4]), and read-based phasing (e.g. WhatsHap [5] and HapCUT2 [6]). Statistical phasing is limited by how well the reference panel represents the sample data and is particularly inaccurate in phasing rare variants [7]. Pedigree-based phasing is accurate for common and rare variants, but pedigree information is typically not available for the inspected individual.

Read-based (or read-aware) phasing works by analyzing sequencing reads that span multiple heterozygous sites to phase them together, resulting in very high accuracy. This method is independent of reference bias, remains unaffected by the rarity of the alleles, and does not require pedigree data. However, since read-based phasing cannot phase regions where heterozygous sites are farther than read sizes, it can only effectively be used in highly variable regions or when using long-reads that span several heterozygous sites.

Here we present TinkerHap, a read-based phasing tool offering consistent performance and enhanced accuracy. TinkerHap excels in accurately handling rare variants and variable genomic regions, such as the Human Leukocyte Antigen (HLA) locus, while also effectively phasing long-read data. Moreover, TinkerHap uniquely integrates information from statistical phasing methods into its read-based framework. This hybrid approach bridges gaps in read coverage and extends haplotype blocks.

Methods

Overview

TinkerHap is implemented in Python 3 and utilizes the *pysam* package [8] for manipulating alignment and variant calling files. The command-line interface accepts an alignment file (SAM/BAM/CRAM) and a variant calling file (VCF/BCF) as inputs, producing a phased VCF file through read-based haplotype phasing. Optionally, TinkerHap can integrate a pre-phased VCF file from a third-party tool (e.g., ShapeIT [1] for statistical-based phasing) to align and merge haplotypes with greater accuracy when possible.

Additionally, TinkerHap can generate multiple output formats to represent the phased haplotypes: a BED file listing the identified haplotype blocks, a BAM file identical to the original but annotated with haplotype and phase information in the Haplotype Phase field (HP) and Haplotype number field (HT), and two separate BAM files - each containing reads corresponding to one of the phased alleles. These outputs facilitate annotation or the splitting of the original alignment into distinct files for each allele, enabling downstream analyses.

Algorithm

TinkerHap implements a three-step phasing algorithm, based on a pairwise distance-based unsupervised classification, designed for precision and scalability. Below is a detailed description of each step with mathematical notations.

1. Identification of Heterozygous Sites

Let $S = \{s_1, s_2, \dots, s_m\}$ represent the set of heterozygous sites identified from the input variant call file (VCF). A site s_i is considered heterozygous if $a_i \neq b_i$ where a_i and b_i are the two alleles at s_i. The loci of these sites are identified as $L(S) = \{l_1, l_2, \dots, l_m\}$, forming the foundation for subsequent phasing steps. Ambiguous allele calls, characterized by low scores

in the VCF's QUAL column and typically caused by sequencing or alignment errors, are flagged.

2. Association of Reads with Heterozygous Sites

Let $R = \{r_1, r_2, \ldots, r_n\}$ denote the set of sequencing reads. Each read r_j spans a subset of heterozygous sites $S_j \subseteq S$. For each read, we map its alleles to the overlapping sites:

$$A(r_j, s_i) = \begin{cases} a_i, & \text{if allele matches reference} \\ b_i, & \text{if allele matches alternative} \end{cases}$$

This step ensures precise allele identification by linking reads to heterozygous sites while accounting for potential alignment errors or ambiguities, such as indels. To ensure accuracy, only reads meeting a minimum mapping quality threshold (e.g., MAPQ ≥ 20) are considered, ensuring that low-confidence alignments do not influence the phasing process.

3. Calculation of Phase Scores

The phasing process begins by arbitrarily assigning the first read r_1 to one of the haplotypes, for instance H_1. This initial assignment acts as a seed to propagate haplotypes across all overlapping reads.

Each read r is then evaluated to determine its phase matching scores $P_1(r)$ and $P_2(r)$ for the two haplotypes, H_1 and H_2. These scores are computed by analyzing all overlapping reads and the heterozygous sites they share with r.

The phase scores are calculated as:

$$P_H(r) = \sum_{k \in K_r} \sum_{s \in S_r \cap S_k} \Delta P_H(r)$$

Where K_r is the set of all overlapping reads for r, and S_r and S_k are the sets of heterozygous sites for reads r and k, respectively. The contribution of each shared site s to the phase score, $\Delta P_H(r)$, is defined as:

$$\Delta P_H(r) = \begin{cases} +w(s), \text{if } A_r(s) = A_k(s) \text{ and } k \text{ belongs to haplotype } H \\ -w(s), \text{if } A_r(s) \neq A_k(s) \text{ and } k \text{ belongs to haplotype } H \end{cases}$$

Here, $A_r(s)$ and $A_k(s)$ represent the alleles of r and k at site s, respectively. The weight $w(s)$ assigned to site s depends on the type of heterozygous site:

$$w(s) = \begin{cases} 2, \text{if } s \text{ is a SNP} \\ 1, \text{if } s \text{ is an indel} \end{cases}$$

This scoring ensures that the phase scores for r are influenced by the agreement or disagreement between r and all overlapping reads at shared heterozygous sites.

After calculating the phase scores, the haplotype HP of r is assigned as follows:

$$\mathrm{HP}(r) = \begin{cases} H_1, \text{if } P_1(r) > P_2(r), \\ H_2, \text{if } P_2(r) > P_1(r), \end{cases}$$

If $P_1(r)$ and $P_2(r)$ are equal, the haplotype assignment can propagate from the overlapping read with the strongest phase connection, or a new haplotype block may be started. This approach ensures consistency in haplotype assignments based on the majority consensus among overlapping reads.

4. Haplotype Extension

Haplotypes are extended iteratively by analyzing overlapping reads. If a read r_k overlaps two or more phased reads $\{r_{j_1}, r_{j_2}, \ldots\}$, its phase is determined by propagating the majority consensus:

$$\mathrm{HP}(r_k) = \{\mathrm{HP}(r_{j_1}), \mathrm{HP}(r_{j_2}), \ldots\}$$

This ensures the consistency of haplotype assignments across contiguous genomic regions. Reads that span conflicting haplotypes are flagged for manual review or downstream quality filtering.

5. Pair-End Read Merging

For paired-end reads (r_i, r_j), the algorithm evaluates the consistency of their haplotypes:

$$M(r_i, r_j) = \begin{cases} +1, & \text{if } \mathrm{HP}(r_i) = \mathrm{HP}(r_j) \\ -1, & \text{if } \mathrm{HP}(r_i) \neq \mathrm{HP}(r_j) \end{cases}$$

Inconsistent pairs trigger a phase reassignment to minimize discordance, leveraging the paired-end linkage information. A weighted graph representation of pair-end links can be constructed for further optimization of haplotype continuity.

6. Integration with Pre-Phased Data (Optional)

When an additional pre-phased VCF file is provided, for example one generated by statistical phasing tools such as ShapeIT, the algorithm merges the read-based haplotypes with the pre-phased data. This involves merging haplotypes and, if necessary, switching the phase numbers (e.g., swapping haplotype 1 and haplotype 2) to ensure consistency with the pre-phased data numbering of haplotypes.

The alignment score $A(b, h)$ for a pre-phased block b and a read-based block h is calculated as:

$$A(b, h) = \sum_{s \in S_b \cap S_h} w(s)$$

where S_b and S_h are the sets of heterozygous sites in b and h, and $w(s)$ represents the weight based on the site type (e.g., SNP or indel). Haplotypes are adjusted to maximize $A(b, h)$,

ensuring that the merged haplotypes align with the pre-phased data and improving the overall phasing accuracy.

7. Output Generation

The final outputs include:

1. Phased VCF: Annotated with a PS (Phase Set) field.

2. Annotated BAM: Each read is tagged with HP (Haplotype Phase) and HT (Haplotype number) fields.

3. Split BAM Files: Separate BAM files for each haplotype, facilitating downstream analyses.

4. BED File: Haplotype boundaries across the genome are defined for visualization.

The detailed algorithm and code can be accessed at: https://github.com/DZeevi-Lab/TinkerHap

Supplementary material for this article, including detailed descriptions of the evaluation and benchmarking procedures, all methodological steps, permissible data, scripts, and resources necessary for reproducibility, is available at https://github.com/DZeevi-Lab/TinkerHap-Supplementary

Evaluation

TinkerHap was evaluated in the following use cases:

1. To evaluate the algorithm's performance in variable regions and for rare variants using Illumina short-reads, we analyzed Whole Genome Sequencing (WGS) data from 1,040 parent-offspring trios that we identified in the UK Biobank [9] on the MHC class II region in humans, specifically on chr6:32,439,878-33,143,325 (hg38 genome version).

2. To evaluate the algorithm's performance with long-reads, we used PacBio long-read sequencing data of the full genomes of GIAB Ashkenazi trio HG002-4 and Chinese trio HG005-7 datasets by Revio (publicly offered by GIAB [10]).

For each offspring in the trios, we constructed a "truth" set of known phased heterozygous sites ("truth sites"). This was achieved by examining loci where each parent possesses different homozygous alleles or where one parent was heterozygous, and the other was homozygous. After preparing the data, we phased the offspring sequence using the following algorithms: ShapeIT[1], WhatsHap[5], HapCUT2[6], TinkerHap, and TinkerHap with ShapeIT[1] phased data as an additional input for merging haplotypes (as described in the "Algorithm" section above). The success rate was evaluated by counting the number of sites in the phased output that matched the truth set.

Results

MHC class II gene region phasing (chr6:32,439,878-33,143,325)

Criteria	TinkerHap+ ShapeIT[1]	TinkerHap	WhatsHap	HapCUT2	ShapeIT
**Phased %**	99.5%	97.1%	86.5%	96.2%	70.5%
**Phasing accuracy %[2]**	96.3%	95.1%	84.9%	94.8%	70.2%
**Phasing accuracy % (SNPs only)**	97.1%	96.0%	86.0%	95.8%	71.6%
**Phasing accuracy % (INDELs only)**	89.6%	87.8%	76.9%	87.4%	59.6%
**Haplotype size (bp)[3]**	21,813	631	75	644	702,123
**Common phased sites errors[4]**	0.14%	0.14%	0.18%	0.12%	0.17%
**Runtime (s)[5]**	6.8	6.5	23.8	9.2	13.7
**Bases / second**	104,005	108,245	29,532	76,105	51,545
**Heterozygous sites / second**	743	773	211	544	368
**Memory usage (MB)**	109	106	397	594	24

Table 1. Phasing performance of different algorithms on short-reads aligned to the MHC class II region

[1] TinkerHap+ShapeIT: TinkerHap algorithm when used with additional ShapeIT pre-phased file.
[2] Phasing accuracy: Successfully phased sites divided by the total number of heterozygous sites.
[3] Haplotype size: Median haplotype size across all samples. “Haplotype” refers to a set of alleles at variant sites along a single chromosome that are inherited together and are guaranteed to be phased together by the algorithm.
[4] Common phased sites errors: Phasing error % in heterozygous sites phased by all algorithms.
[5] Runtime: Median runtime per sample.

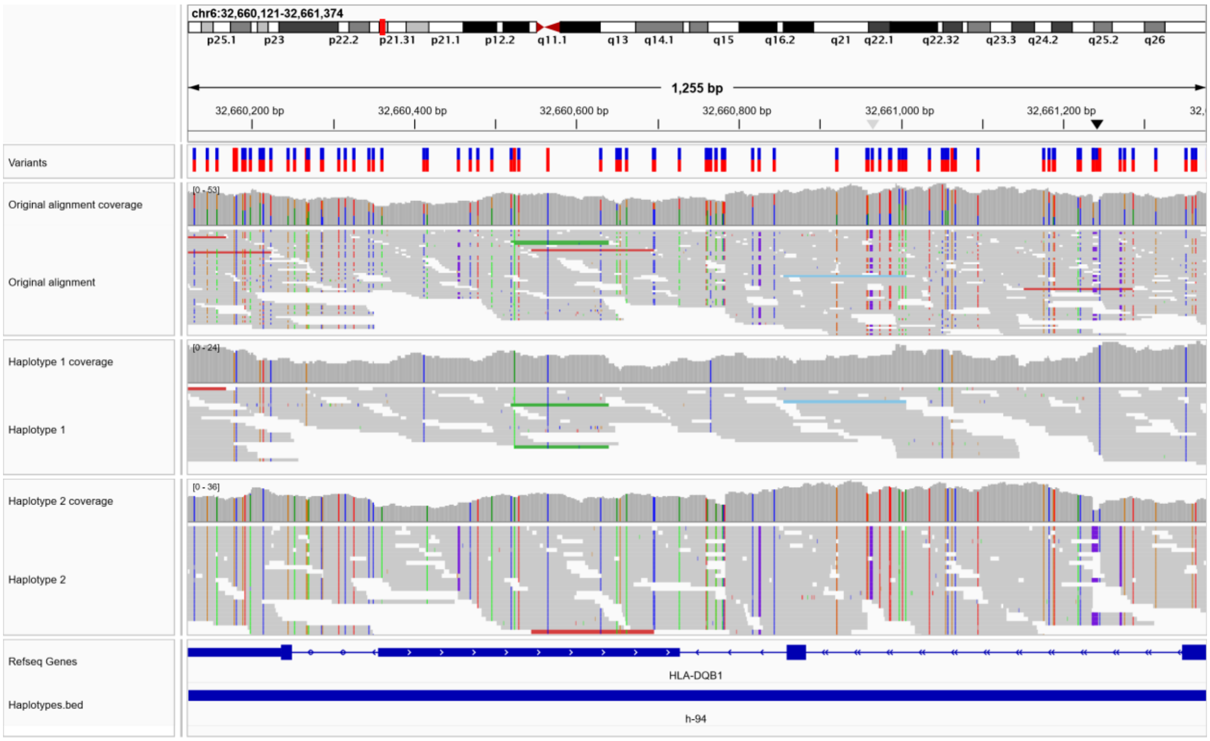

Figure 1. Phased BAM Outputs displaying Heterozygous sites (IGV [11] screenshot) - The upper track is the original alignment, while the two tracks below represent the output of Haplotype 1 and Haplotype 2. Heterozygous sites are correctly segregated between the two phases, demonstrating successful phasing. The continuous blue line in the bottom track illustrates a BED file annotation, highlighting the size of the haplotype region where all variants are confirmed to share the same phase.

PacBio phasing (whole genome)

Criteria	TinkerHap	WhatsHap	HapCUT2
**Phased %**	99.4%	96.8%	96.7%
**Phasing accuracy %[1]**	97.5%	95.5%	95.4%
**Phasing accuracy % (SNPs only)**	97.8%	96.5%	96.4%
**Phasing accuracy % (INDELs only)**	96.0%	90.2%	90.5%
**Haplotype size (bp)[2]**	79,449	68,303	72,220
**Common phased sites errors[3]**	0.47%	0.47%	0.55%
**Runtime (s)[4]**	10,519	17,578	5,495
**Bases / second**	298,239	178,475	570,891
**Heterozygous sites / second**	433	259	829

**Memory usage (MB)**	1,726	1,696	876

Table 2. Phasing performance of different algorithms on long-reads of full genomes.
[1] Phasing accuracy: Successfully phased sites divided by the total number of heterozygous sites.
[2] Haplotype size: Median haplotype size across all samples.
[3] Common phased sites errors: Phasing error % in heterozygous sites phased by all algorithms.
[4] Runtime: Median runtime per sample.

Discussion

Here, we introduce TinkerHap, a read-based phasing algorithm designed for accurate and reliable phasing across diverse genomic contexts, with the ability to integrate statistical phasing data from third-party tools for improved performance.

We evaluated TinkerHap using two datasets: the MHC class II region in humans with Illumina short-read WGS data to assess its accuracy in variable regions, and PacBio sequencing data to evaluate its performance with long-reads. These datasets were selected due to their suitability for testing read-based phasing algorithms, as both are characterized by a high density of variants that provide many opportunities for phasing.

Performance of Short-Reads in Variable Regions

In the MHC class II region using short-reads, TinkerHap phased 97.1% of variants with 95.1% accuracy. In comparison, the second-best algorithm phased 96.2% of variants with 94.8% accuracy. All methods showed higher phasing accuracy for SNPs compared to indels (97.1% and 89.6%, respectively, in TinkerHap).

Performance of Long-Read Sequencing

TinkerHap achieved a phasing accuracy of 97.5% for SNPs and 96.0% for indels with PacBio datasets. These results were superior to the second-best algorithm, which demonstrated accuracies of 95.5% and 95.4%, respectively. Moreover, TinkerHap produced longer

haplotype blocks (median size: 79,449 bp) compared to the second-best algorithm (68,303 bp). Runtime analysis revealed that TinkerHap required 10,519 seconds per sample, compared to 5,495 seconds for the fastest algorithm.

Comparison of Long-Read and Short-Read Performance

TinkerHap performed better with long-read sequencing data compared to short-read data in several key metrics. Long-reads offer superior upstream alignment quality, particularly at highly variable sites, which enhances the overall accuracy of variant calling and subsequent phasing steps. Long-read data yielded more extensive haplotype blocks (median size: 79,449 bp compared to 631 bp with short-reads) and higher phasing accuracy (97.5% for SNPs in long-reads compared to 96.0% in short-reads, and 96% for indels in long-reads compared to 87.8% in short-reads). This improved performance is expected due to long-reads containing more heterozygote sites and enabling improved alignments.

Integration with Statistical Phasing

TinkerHap uniquely includes the ability to integrate data from third-party tools, such as ShapeIT. By incorporating pre-phased haplotypes, the TinkerHap + ShapeIT combination achieved 99.5% phased variants with 96.3% accuracy, significantly outperforming standalone methods. This hybrid approach improved haplotype block continuity and effectively addressed gaps in read coverage.

Limitations

TinkerHap’s runtime and memory usage for long-read data present areas for potential optimization, and it currently lacks support for polyploid genomes. TinkerHap is limited in merging distant haplotypes, which could be particularly useful for applications such as exome sequencing. Future incorporation of pedigree information could address this issue and enhance TinkerHap’s accuracy in trio or family-based studies.

In most phasing errors that we manually examined, inaccuracies were primarily attributed to upstream variant calling rather than to the phasing algorithm itself. This suggests that TinkerHap may be approaching the limit of what can be achieved with downstream read-based phasing alone. This underscores the importance of high-quality preprocessing.

Availability of supporting source code and requirements

Project name: TinkerHap

Project home page: https://github.com/DZeevi-Lab/TinkerHap

Supplementary information: https://github.com/DZeevi-Lab/TinkerHap-Supplementary

Operating system(s): Platform independent

Programming language: Python

Other requirements: Python 3.6.0 or higher, pysam 0.17 or higher

License: MIT

Data availability

UK Biobank data: The Illumina short-read data from 1,040 parent-offspring trios used in this study were accessed from the UK Biobank under application number 74655. These data are available under controlled access due to participant privacy considerations. Researchers can apply for access through the UK Biobank Access Management System by submitting a detailed research proposal. Further information and application guidelines are available at https://www.ukbiobank.ac.uk/enable-your-research/apply-for-access.

Genome in a Bottle (GIAB) data: The long-read sequencing data from the GIAB Ashkenazi and Chinese trios used for algorithm evaluation are publicly available at the National Center

for Biotechnology Information (NCBI) FTP site. The datasets can be accessed directly via https://ftp-trace.ncbi.nlm.nih.gov/ReferenceSamples/giab/data/. Further information is available in the supplementary material for this article.

Funding

This research was supported by the ISRAEL SCIENCE FOUNDATION and JDRF (grant No. 2658/21).

Competing interests

The authors declare no competing interests.

Author contribution:

Conceptualization – U.H., D.Z.; Methodology - U.H.; Formal Analysis - U.H.; Investigation - U.H; Writing, original draft preparation – U.H.; Writing, review & editing - U.H., D.Z., E.S., D.N, I.B.; Visualization - U.H. Supervision – D.Z.; Funding Acquisition – D.Z.

Acknowledgements:

We thank Rona Gershon Talmi from the Hamaabada Podcast (Kan) and Dr. Jeremy Fogel and Tuval Rosenwasser from the Think & Drink Different Podcast for their contribution to this work.

This research has been conducted using the UK Biobank Resource under application number 74655.

References

[1] O. Delaneau, J. Marchini, and J. F. Zagury, “A linear complexity phasing method for thousands of genomes,” *Nature Methods 2011 9:2*, vol. 9, no. 2, pp. 179–181, Dec. 2011, doi: 10.1038/nmeth.1785.

[2] B. L. Browning, X. Tian, Y. Zhou, and S. R. Browning, “Fast two-stage phasing of large-scale sequence data,” *The American Journal of Human Genetics*, vol. 108, no. 10, pp. 1880–1890, Oct. 2021, doi: 10.1016/j.ajhg.2021.08.005.

[3] T. Druet and M. Georges, “LINKPHASE3: an improved pedigree-based phasing algorithm robust to genotyping and map errors,” *Bioinformatics*, vol. 31, no. 10, pp. 1677–1679, May 2015, doi: 10.1093/bioinformatics/btu859.

[4] D. B. Miller and S. R. Piccolo, “trioPhaser: using Mendelian inheritance logic to improve genomic phasing of trios,” *BMC Bioinformatics*, vol. 22, no. 1, p. 559, Dec. 2021, doi: 10.1186/s12859-021-04470-4.

[5] M. Martin *et al.*, “WhatsHap: fast and accurate read-based phasing,” *bioRxiv*, p. 085050, Nov. 2016, doi: 10.1101/085050.

[6] V. Bansal, “HapCUT2: A Method for Phasing Genomes Using Experimental Sequence Data,” *Methods in Molecular Biology*, vol. 2590, pp. 139–147, 2023, doi: 10.1007/978-1-0716-2819-5_9.

[7] Y. Choi, A. P. Chan, E. Kirkness, A. Telenti, and N. J. Schork, “Comparison of phasing strategies for whole human genomes,” *PLoS Genet*, vol. 14, no. 4, p. e1007308, Apr. 2018, doi: 10.1371/journal.pgen.1007308.

[8] A. Heger, “Pysam: HTSlib interface for Python, 2009,” https://github.com/pysam-developers/pysam.

[9] C. Sudlow *et al.*, “UK Biobank: An Open Access Resource for Identifying the Causes of a Wide Range of Complex Diseases of Middle and Old Age,” *PLoS Med*, vol. 12, no. 3, p. 1001779, Mar. 2015, doi: 10.1371/JOURNAL.PMED.1001779.

[10] Genome in a Bottle Consortium, “GIAB Benchmarking Data,” 2023, Accessed: Dec. 12, 2024. [Online]. Available: https://ftp-trace.ncbi.nlm.nih.gov/ReferenceSamples/giab/data/

[11] H. Thorvaldsdottir, J. T. Robinson, and J. P. Mesirov, “Integrative Genomics Viewer (IGV): high-performance genomics data visualization and exploration,” *Brief Bioinform*, vol. 14, no. 2, pp. 178–192, Mar. 2013, doi: 10.1093/bib/bbs017.

Click here to access/download
Supplementary Material
TinkerHap-Supplementary-20250518.docx

Personal Cover Click here to access/download;Personal Cover;personal-cover.docx

May 18, 2025

Dear Editor,

I am writing on behalf of my coauthors to submit our manuscript, "TinkerHap – A Novel Read-Based Phasing Algorithm with Integrated Multi-Method Support for Enhanced Accuracy," for consideration as a Technical Note in GigaScience.

In this work we developed a novel and unique algorithm for phasing. Phasing is the assignment of alleles to their respective parental chromosomes. It is crucial for our understanding of how certain allelic combinations drive disease risk, especially in complex traits.

Various methods of phasing are currently available, but each suffers significant inaccuracies and challenges when applied alone. Statistical phasing methods are limited by how well a reference panel represents the sample data and are particularly inaccurate in phasing rare variants (which are the most important variants for identifying the genetic basis of diseases). Pedigree-based phasing is typically not available for an inspected individual, and read-based phasing is limited in regions with sparse genetic variation (especially coding regions) and produces short haplotype blocks.

Our method goes beyond traditional phasing approaches by integrating different methods together in several steps.

For the first step we developed a new read-based phaser, based on a pairwise distance-based unsupervised classification. We then combined our read-based algorithm with externally phased data, such as statistical or pedigree phasing, to improve the accuracy of the phasing and increase the length of the haplotype blocks. This hybrid approach resulted in significant improvement of phasing over stand-alone algorithms. We tested TinkerHap hybrid algorithm and three other algorithms (WhatsHap, HapCUT2 and ShapeIT) on UK Biobank trios on the MHC class 2 genomic region that was sequenced with Illumina short reads. Hybrid TinkerHap was able to phase 99.5% of heterozygote sites, with 96.3% accuracy and 21,831 bps mean haplotype block length. HapCUT2, the second best algorithm, phased 96.2% of heterozygote sites, at 94.8% accuracy and with mean haplotype block size of only 644 bps. ShapeIT, which is a stand-alone statistical based phaser was able to phase only 70.5% of the heterozygote sites.

As the median gene size in the human genome is approximately 24,000 bps, TinkerHap is the first phasing algorithm that can phase entire genes with extremely high accuracy. This is a crucial advancement for studies of the genetic basis of traits and diseases, as it allows to accurately infer the two full human protein products of a gene from DNA sequencing.

Lastly, the recent shift to whole genome sequencing (WGS) in biobanks (e.g. the UK Biobank published the entire cohort 500,000 whole genomes approximately a year ago) offers an unprecedented opportunity for large-scale phasing that can serve multiple researchers in studying

multiple diseases and traits. An accurate phasing algorithm with the ability to phase large haplotype blocks will enable researchers to uncover complex patterns of inheritance and identify how groups of phased variants interact to cause complex diseases.

All authors have approved this manuscript submission for publication. There are no conflicts of interest to declare. We are grateful for your consideration of our work and would welcome the opportunity to provide any additional information that might assist the review process.

Thank you for your time and consideration. We look forward to your response.

Sincerely,

Danny Zeevi, PhD

Chair, Department of Biotechnology

Jerusalem Multidisciplinary College, Jerusalem, Israel
