# Supplementary material for: TinkerHap—a novel read-based phasing algorithm with integrated multimethod support for enhanced accuracy
Source: Gigascience. 2025 Oct 28;14:giaf138. doi: 10.1093/gigascience/giaf138 (PMC12723663; doi:10.1093/gigascience/giaf138)
Supplement: giaf138_GIGA-D-25-00150_Revision_1 [file giaf138_giga-d-25-00150_revision_1.pdf]

GigaScience

TinkerHap - A Novel Read-Based Phasing Algorithm with Integrated Multi-Method Support for Enhanced Accuracy

--Manuscript Draft--

**Manuscript Number:**	GIGA-D-25-00150R1
**Full Title:**	TinkerHap - A Novel Read-Based Phasing Algorithm with Integrated Multi-Method Support for Enhanced Accuracy
**Article Type:**	Technical Note
**Funding Information:**	Juvenile Diabetes Research Foundation in Israel (2658/21) — Dr. Danny Zeevi<br>Israel Science Foundation (2658/21) — Dr. Danny Zeevi
**Abstract:**	Phasing, the assignment of alleles to their respective parental chromosomes, is fundamental to studying genetic variation and identifying disease-causing variants. Traditional approaches, including statistical, pedigree-based, and read-based phasing, face challenges such as limited accuracy for rare variants, reliance on external reference panels, and constraints in regions with sparse genetic variation.<br>To address these limitations, we developed TinkerHap, a novel phasing algorithm that integrates a read-based phaser, based on a pairwise distance-based unsupervised classification, with external phased data, such as statistical or pedigree phasing. We evaluated TinkerHap's performance against other phasing algorithms using 1,040 parent-offspring trios from the UK Biobank (Illumina short-reads) and GIAB Ashkenazi trio (PacBio long-reads). TinkerHap's read-based phaser alone achieved higher phasing accuracies than all other algorithms with 95.1% for short-reads (second best: 94.8%) and 97.5% for long-reads (second best: 95.5%). Its hybrid approach further enhanced short-read performance to 96.3% accuracy and was able to phase 99.5% of all heterozygous sites. TinkerHap also extended haplotype block sizes to a median of 79,449 base-pairs for long-reads (second best: 68,303 bp) and demonstrated higher accuracy for both SNPs and indels. This combination of a robust read-based algorithm and hybrid strategy makes TinkerHap a uniquely powerful tool for genomic analyses.
**Corresponding Author:**	Uri Hartmann<br>Hadassah Academic College<br>Jerusalem, ISRAEL
**Corresponding Author Secondary Information:**	
**Corresponding Author's Institution:**	Hadassah Academic College
**Corresponding Author's Secondary Institution:**	
**First Author:**	Uri Hartmann
**First Author Secondary Information:**	
**Order of Authors:**	Uri Hartmann<br>Eran Shaham<br>Dafna Nathan<br>Ilana Blech<br>Danny Zeevi
**Order of Authors Secondary Information:**	
**Response to Reviewers:**	(stylized response letter is attached separately)<br><br>We would like to thank you and the reviewers for the constructive and thoughtful peer review of our manuscript. We are hereby submitting the revised version of the

Powered by Editorial Manager® and ProduXion Manager® from Aries Systems Corporation

manuscript along with the supplementary material. In addition, we have uploaded to the SFTP server the relevant files associated with the additions of TinkerHap+ShapeIT and ShapeIT, and we have also updated the TinkerHap-Supplementary repository on GitHub.
Email points:
"One important topic mentioned in the reports is the need to present an improved and fair assessment of the method, also with a wider scope, e.g. on a chromosome-/ genome-wide scale"

We agree that this is a very important point. Therefore, in the second part of the analysis we included a genome-wide evaluation of our method on samples from the GIAB project, which provides a comprehensive benchmark across the full genome. It is important to note that such analysis is relevant only when using long reads. Since short sequencing reads are typically ~150 bp long, and variants are typically 500-1000 bp apart, individual sequencing reads rarely cover more than one variant, so any read-based phasing method such as TinkerHap would not produce additional or meaningful phasing information. This is why we focused on our short-read analysis on the MHC class II region which is the most variable in the genome.
To emphasize these limitations of our method and make it clearer to the readers, we added the following paragraph to the discussion section:
"A key limitation of read-based phasing approaches, including TinkerHap, arises from the typical length of short sequencing reads (~150 bp) relative to the spacing between variants (500-1,000 bp on average). As a result, individual reads seldom span multiple variants, thereby yielding little to no additional or informative phasing data. This limitation confines the effective application of read-based phasing methods like TinkerHap primarily to long-read sequencing projects, where reads are sufficiently extended to span multiple variants, or to genomic regions exhibiting high variant density (greater than approximately 1 variant per 100 bp), such as the human leukocyte antigen (HLA) locus."
"In addition to addressing the reviewers' comments, please register any new software application in the bio.tools and SciCrunch.org databases to receive RRID (Research Resource Identification Initiative ID) and biotoolsID identifiers, and include these in your manuscript. Computational workflows should be registered in workflowhub.eu and the DOIs cited in the relevant places in the manuscript. These will facilitate tracking, reproducibility and re-use of your tool."

We thank you and the reviewers for pointing out these important databases. We have registered TinkerHap in the bio.tools and SciCrunch.org databases, and the manuscript now includes the assigned biotoolsID and RRID identifiers to facilitate tracking, reproducibility, and reuse of the tool. Regarding WorkflowHub.eu, we believe this registry is less applicable to TinkerHap, as TinkerHap is a standalone software tool for read-based variant phasing rather than a multi-step computational workflow.

Reviewer reports:
Reviewer #1.1: "The benchmarking was only performed on MHC Class II, which is a relatively small and easy to phase region based on the high level of heterozygosity. How does the statistics look when applied to the whole genome? After generating the phased read set, what % of reads can be accurately assigned to the original haplotype in the whole genome scale? To benchmark the latter, I would recommend doing it on HG002 phased variants and reads by using the HG002Q100 genome (https://github.com/marbl/hg002) - i.e. map the classified reads and calculate the coverage and accuracy based on where the reads align to. I would be curious to see how the MHC Class II phased read alignment looks like on the HG002Q100 truth assembly, on each haplotype."

Thank you very much for your thoughtful comments and suggestions.
Regarding the benchmarking on MHC Class II, we acknowledge that this region benefits from high heterozygosity, making it particularly suitable for phasing. We agree that it is essential to test TinkerHap on a genome-wide scale. Therefore, we have already analyzed TinkerHap's performance on the whole-genome scale for HG002 and an additional GIAB sample, with the results presented in Table 2.
For short-reads, since short sequencing reads are typically ~150 bp long, and variants are typically 500-1000 bp apart, individual sequencing reads rarely cover more than

Powered by Editorial Manager® and ProduXion Manager® from Aries Systems Corporation

one variant, so applying any read-based phasing method such as TinkerHap on short reads would not produce additional or meaningful phasing information to many regions of the genome. To address this, we have now added a caveat to the Discussion section highlighting this limitation of read-based phasing methods like TinkerHap:
"A key limitation of read-based phasing approaches, including TinkerHap, arises from the typical length of short sequencing reads (~150 bp) relative to the spacing between variants (500-1,000 bp on average). As a result, individual reads seldom span multiple variants, thereby yielding little to no additional or informative phasing data. This limitation confines the effective application of read-based phasing methods like TinkerHap primarily to long-read sequencing projects, where reads are sufficiently extended to span multiple variants, or to genomic regions exhibiting high variant density (greater than approximately 1 variant per 100 bp), such as the human leukocyte antigen (HLA) locus."

Regarding the MHC Class II phased read alignment performance on truth assembly such as HG002Q100, it is important to note that we benchmarked TinkerHap on approximately 1,000 diverse short-read samples of trios, with truth phasing learned from parent-offspring inheritance.
Reviewer #1.2: "When showing benchmarking results, key features are missing - 1) number of heterozygous variant sites are used for phasing, in addition to the Phased % (what's the denominator here?), 2) number of phase blocks, phase block NG50 and total length and 3) Show the NGx length distribution by plotting the cumulative covered genome length as a function of the longest to shortest phase block."
We agree, and added the number of heterozygous variant sites used for phasing to the manuscript as well as the number of phase blocks, phase block N50 and the total length of the phased blocks.
Reviewer #1.3: "After phasing the variants (and reads), are the authors accurately able to type the HLA Class II genes? The goal of MHC phasing is to accurately genotype the HLA-genes. It is unclear to me why the authors applied their phasing on the 1,040 parent-offspring trios. I agree that it is 'phasable', however, it is unclear what the motivation here is - the MHC Class II is particularly known to have linked HLA types (e.g., HLA-DRB3 and HLA-DRB5 are inherited together depending on the HLA-DRB1 type, while in some haplotypes HLA-DRB3 is entirely missing), and depending on the HLA types and because the reference is incompletely representing this locus, there are multiple tools developed for genotyping this locus. I would be more convinced if the authors could show the HLA genotyping accuracy together based on their phasing method."
We agree that accurate typing of HLA Class II genes is very important. In this work we chose to evaluate phasing performance based on truth datasets learned from trios, which is the golden standard for phasing evaluation. Testing the accuracy of phasing based on the accuracy of typing might introduce biases due to the different methods the reference typing sets were created by.
Reviewer #1.4: "Is it possible to use additional data types to further extend the phase blocks, by using datasets such as low coverage PacBio data in addition to the short-read WGS? How about phasing with linked-reads or Hi-C? Both Whatshap and HapCut2 are specifically designed to combine such short and long-range datasets, giving the advantage of using such tools."
We agree that long reads such as PacBio and other techniques such as Hi-C can contribute to evaluating TinkerHap performance and to extend phasing blocks. Therefore, we have analyzed TinkerHap's performance on PacBio long reads and the results are presented in Table 2. We did not have access to Hi-C linked reads in combination with its raw data sequencing reads, and we believe that such broader comparison that integrates multiple additional technologies and tools would be better suited to a larger dedicated study, and we view this as an interesting direction for future work.
Reviewer #1.5: "The authors claim their method is free from reference bias, which I strongly disagree. Using a bam file aligned to a reference inherently has the issue of mapping biases, so any such tools are limited by the reads that aligns incorrectly. Repeats, especially copy number variable region with collapses in the reference are very difficult to accurately phase. Any large structural variant not properly represented in the reference will cause problems due to unmapped reads."
Very good point, the differentiation between reference bias in multiple phased genomes and reference bias of the reference genome was not clear in our manuscript. The manuscript has been revised to explain that the method itself is free from statistical

bias, however mapping bias from reference-based alignment remains a known limitation

Reviewer #1.6: "In Methods, 2nd section - I would suggest to use allele 1 and allele 2 instead of 'reference' and 'alternative' in the equation and the code. This will increase the number of heterozygous 'phasable' variants that does not carry any reference allele."

We thank the reviewer for this excellent suggestion. The manuscript and corresponding code have been updated to use "allele 1" and "allele 2" instead of "reference" and "alternative" in the relevant section.

Reviewer #2.1: "The authors are missing experiments for long-read based phasing. How does TinkerHap performs with ShapeIT on PacBio long-reads? I would suggest the authors using the same phasing method class as their short-read analysis: TinkerHap+ShapeIT; TinkerHap; WhatsHap; HapCUT2; ShapeIT. Also I believe ShapeIT is capable to take long-read SNV/INDEL calls as vcf."

We thank the reviewer for this valuable comment and fully agree with the suggestion. In response, we have extended our PacBio long-read benchmarking to include both TinkerHap+ShapeIT and ShapeIT alone, alongside the other methods. These additions provide a more complete and balanced evaluation of the different phasing strategies, and the results are now included in Table 2 of the revised manuscript.

Reviewer #2.2: "Following up on the point 1, the experimental design of this study is quite skewed. WhatsHap is not suitable for short-read sequencing data. It does not make sense to apply WhatsHap on short-read data."

This is a very good point. We compared WhatsHap on PacBio long reads (table 2), and used it for short reads only on the MHC class II region, because this region contains sufficient variant density for effective read-based phasing even with short-reads, making it comparable in applicability to TinkerHap. We have now also added the number of heterozygous variant sites used for phasing, which allows the reader a better understanding of the framework that the different methods were compared in

Reviewer #2.3: "I would caution the authors to read and potentially compare with SAPPHIRE (https://doi.org/10.1371/journal.pgen.1011092). This is a method that developed by the ShapeIT team for incorporating long-read sequencing data and ShapeIT. "

SAPPHIRE is a great tool for phasing, that includes ancestry-aware phasing of large-scale genotype datasets. However it does not accept a .bam or .cram alignment files as input, and does not include a read-based feature. In this work we focused on benchmarking against well-established and widely adopted read-based phasing methods to provide a stable and recognized reference frame. Combining TinkerHap with additional categories of phasing tools in order to get better phasing is a great option for future work.

Reviewer #2.4: "To better justify the hybrid strategy, I recommend adding an analysis of sites where TinkerHap and ShapeIT disagree. Are these differences due to reference bias, read coverage, variant type, or true ambiguity? Such an evaluation would help users understand when to rely on the read-based output vs. ShapeIT, and enhance confidence in the merging strategy."

An in-depth disagreement analysis should be very interesting and insightful. Since in many cases the determination of the reason for discrepancy might be impossible to do with certainty and can cause bias in the analysis, the major difference between ShapeIT and TinkerHap is that ShapeIT (and other statistical phasing algorithms) is less likely to accurately phase rare variants, while TinkerHap (and other read-based algorithms) are not affected by the frequency of the variant, but rather by the density of the variants and length of reads. For example, a previous study has shown that for ShapeIT, the switch error rate (SER) increases as the minor allele frequency (MAF) decreases, particularly for MAF below 1% (Choi et al., 2018).

To make it clearer to the readers, we have added to the manuscript the following paragraph in the discussion:

"An important advantage of TinkerHap, as well as all read-based algorithms, is that it is less likely to be affected by the rarity of a variant (Choi et al., 2018). However, the density of the variants in combination with the length of the sequencing read are key factors for the performance of TinkerHap. A key limitation of read-based phasing approaches, including TinkerHap, arises from the typical length of short sequencing reads (~150 bp) relative to the spacing between variants (500-1,000 bp on average). As a result, individual reads seldom span multiple variants, thereby yielding little to no additional or informative phasing data. This limitation confines the effective application of read-based phasing methods like TinkerHap primarily to long-read sequencing

Powered by Editorial Manager® and ProduXion Manager® from Aries Systems Corporation

projects, where reads are sufficiently extended to span multiple variants, or to genomic regions exhibiting high variant density (greater than approximately 1 variant per 100 bp), such as the human leukocyte antigen (HLA) locus."

Reviewer #2.5: "I could see the versions of the software in the supplementary github, but I think it is also important to include those in the manuscript. For example, shapeIT 2-5 are having quite different functions. The citation for ShapeIT in the manuscript is for ShapeIT 2, but the program that has been used is for ShapeIT 5. "
Thank you for noticing this. The manuscript has been revised so that the citation now correctly matches the version of ShapeIT that was used.
Reviewer #2.6: "Need to mention the benchmarking hardware information for runtime comparison."
Thank you for pointing this out. The benchmarking hardware information was already included in the supplementary material, but we now added it also to the main manuscript.
Reviewer #2.7: ""...a novel and unique phasing algorithm..." -> "...a novel phasing algorithm..."
Thank you for ponting this out - The wording has been updated accordingly in the manuscript.
Reviewer #3.1: "Limited scope of benchmarking
The evaluation on the highly polymorphic MHC class II region is appropriate for highlighting TinkerHap's strengths in phasing rare variants in variable regions. However, the current evaluation on short -read based phasing is based on a ~700 kb region selected for its high variant density, which limits the generalizability of the findings. Since the manuscript emphasizes improved performance in regions with sparse genetic variation, it would strengthen the work to include chromosome-wide or genome-wide benchmarks, particularly on short-read data. This would also provide a more balanced comparison with tools like SHAPEIT5, which predictably underperform in the MHC class II region due to their reliance on population allele frequencies and linkage disequilibrium patterns that are less effective for rare or private variants."
Thank you very much for your thoughtful comments and suggestions.
Regarding the benchmarking on MHC Class II, we acknowledge that this region benefits from high heterozygosity, making it particularly suitable for phasing. We agree that it is essential to test TinkerHap on a genome-wide scale. Therefore, we have already analyzed TinkerHap's performance on the whole-genome scale on GIAB samples, with the results presented in Table 2.
For short-reads, since short sequencing reads are typically ~150 bp long, and variants are typically 500-1000 bp apart on average in the genome, individual sequencing reads rarely cover more than one variant, so applying any read-based phasing method such as TinkerHap on short reads would not produce additional or meaningful phasing information to most regions of the genome. To address this, we have now added a caveat to the Discussion section highlighting this limitation of read-based phasing methods like TinkerHap:
"A key limitation of read-based phasing approaches, including TinkerHap, arises from the typical length of short sequencing reads (~150 bp) relative to the spacing between variants (500-1,000 bp on average). As a result, individual reads seldom span multiple variants, thereby yielding little to no additional or informative phasing data. This limitation confines the effective application of read-based phasing methods like TinkerHap primarily to long-read sequencing projects, where reads are sufficiently extended to span multiple variants, or to genomic regions exhibiting high variant density (greater than approximately 1 variant per 100 bp), such as the human leukocyte antigen (HLA) locus."
We have also revised the text to remove the reference to "sparse genetic variation".
Reviewer #3.2: "Coverage and scalability
The manuscript describes TinkerHap as scalable, but since the algorithm relies on overlapping reads, it is unclear how its performance varies with sequencing depth. Including a figure or supplementary analysis showing phasing accuracy, runtime, and memory usage at different coverage levels (particularly for short-read data) would help support this claim and guide users on appropriate coverage requirements."
Thank you pointing this out. The manuscript includes comparisons of runtime and memory usage, which offer practical insights into the tool's scalability relative to other methods. To prevent any overstatement, we have removed the phrase "and scalability" from the algorithm description.
Reviewer #3.3: "Clarify algorithmic novelty

It would be helpful to elaborate on how TinkerHap's read-based phasing algorithm differs from existing approaches such as the weighted Minimum Error Correction (wMEC) framework implemented in WhatsHap. For example, what specifically enables TinkerHap's read-based mode to produce longer haplotype blocks than other read-based tools?"
The key difference between TinkerHap and existing approaches such as WhatsHap lies in the algorithmic strategy used to address this NP-hard problem. WhatsHap's runtime complexity is O(2k m), where k is the maximum read coverage at any variant site, and m is the number of SNP positions. To avoid exponential runtime, WhatsHap enforces an internal coverage cap (flag: "--internal-downsampling"), with a default k=15 and a hard limit of k=23. As shown in Figure 2 of the WhatsHap paper, reducing coverage increases both the error rate and the proportion of unphasable positions.
In our experiments, we used data from the UK BioBank. This data has an average coverage of x32.5. At this depth, WhatsHap would either (i) run with an unfeasible runtime if coverage is not capped, or (ii) require downsampling, leading to higher error rates and more unphasable positions.
In contrast, TinkerHap formulates read-based phasing as a K=2 clustering problem. It takes an iterative greedy expansion approach, with an overall polynomial runtime. Like other clustering algorithms (e.g., K-Means), TinkerHap is sensitive to initialization. However, this can be mitigated with multiple runs with different initializations.
To conclude, TinkerHap enjoys a polynomial runtime, while harnessing the full benefits of a high coverage to produce longer haplotype blocks with fewer errors and unphasable sites.
Reviewer #3.4: "Data description
A brief characterization of the input datasets, such as the sequencing depth, as well as the number and average genomic distance of heterozygous variants in the MHC class II region and the GIAB trio data would provide important context for interpreting the reported phasing accuracy and haplotype block lengths."
- We agree it is very important, and so sequencing depth and the number of heterozygous variant sites used for phasing have now been added to the manuscript.
Reviewer #3.5: "Manuscript structure
Since the algorithm itself is the core novel contribution, it should be part of the results section, as well as the description of the evaluation currently in placed in the discussion. According to GigaScience's Technical Note guidelines, the method section should be reserved for "any additional methods used in the manuscript, that are not part of the new work being described in the manuscript."
Thank you very much for your thoughtful suggestion regarding the manuscript's structure and for referencing GigaScience's Technical Note guidelines. We greatly appreciate your attention to detail in ensuring alignment with journal standards.
Upon careful review of the guidelines, we recognize that the Methods section is intended for additional methods not part of the new work, while the Findings section should detail the implementation, availability, and testing of the novel contribution in a reproducible manner. In our manuscript, we placed the TinkerHap algorithm description in Methods to provide a dedicated space for the technical details needed for reproduction, as this aligns with another requirement of the technical guidelines for the method section - "This section should provide enough detail to allow other researchers to interpret and repeat the study." We also think that conventional expectations in bioinformatics papers are that researchers often look to Methods for algorithmic specifics to interpret and repeat the work, while focusing on the results section for evaluation and benchmarking.
To better adhere to the guidelines without major restructuring, we have added cross-references and a subsection in Results explicitly linking to the algorithm's implementation details, emphasizing reproducibility. ~Based on the algorithm described in the Methods section...~
Reviewer #3.6: "Novelty of hybrid approach
While TinkerHap's ability to integrate externally phased haplotypes is valuable, similar functionality exists in other tools, for example, SHAPEIT can accept pre-phased scaffolds (including those generated from read-based phasing), and WhatsHap supports trio-based phasing. Consider refining the language to more precisely describe what is uniquely implemented in TinkerHap's hybrid strategy. It would be interesting to see how the presented results of using SHAPEIT's phasing output as input for TinkerHap compare to an approach of feeding TinkerHap's read-based phasing results into SHAPEIT."
Great point. The introduction text now specifies that TinkerHap uniquely merges

Powered by Editorial Manager® and ProduXion Manager® from Aries Systems Corporation

	external phasing blocks into its read based framework while maintaining block continuity, which differs from existing tools that do not integrate merging within a read aware propagation step.<br>Reviewer #3.7: "Reference bias claim<br>The introduction states that read-based phasing is "independent of reference bias." While this approach is generally less susceptible to reference bias than statistical phasing, bias can still arise during the read alignment stage, potentially affecting downstream phasing. This point should be clarified."<br>Great point, the differentiation between reference bias in multiple phased genomes and reference bias of the reference genome was not clear in our manuscript.- The manuscript has been revised to explain that the method itself is free from statistical bias, however mapping bias from reference-based alignment remains a known limitation<br>Reviewer #3.8: "GIAB datasets<br>The abstract mentions only the GIAB Ashkenazi trio, but later the Chinese trio is included in the analysis as well. Please clarify whether results are averaged across the two datasets."<br>Thank you for noticing this. The manuscript now states that results are averaged across the Ashkenazi and Chinese GIAB trios.<br>Reviewer #3.19 " Tool version citation<br>Please clarify in the text that the comparison was made using SHAPEIT5, not an earlier version."<br>Thank you for noticing this. The manuscript has been revised to clearly state that all benchmarking was performed using ShapeIT5, and the citation now matches this version.
**Additional Information:**	
**Question**	**Response**
Are you submitting this manuscript to a special series or article collection?	No
**Experimental design and statistics**<br><br>Full details of the experimental design and statistical methods used should be given in the Methods section, as detailed in our Minimum Standards Reporting Checklist. Information essential to interpreting the data presented should be made available in the figure legends.<br><br>Have you included all the information requested in your manuscript?	Yes
**Resources**<br><br>A description of all resources used, including antibodies, cell lines, animals and software tools, with enough information to allow them to be uniquely identified, should be included in the Methods section. Authors are strongly encouraged to cite Research Resource Identifiers (RRIDs) for antibodies, model	Yes

organisms and tools, where possible.<br><br>Have you included the information requested as detailed in our Minimum Standards Reporting Checklist?	
**Availability of data and materials**<br><br>All datasets and code on which the conclusions of the paper rely must be either included in your submission or deposited in publicly available repositories (where available and ethically appropriate), referencing such data using a unique identifier in the references and in the “Availability of Data and Materials” section of your manuscript.<br><br>Have you have met the above requirement as detailed in our Minimum Standards Reporting Checklist?	Yes
GigaScience has policies and guidelines in place for the use of generative AI-writing tools such as ChatGPT. If you have used such writing tools to assist with writing the manuscript this must be declared and cited in the text. Authors should not list AI-writing tools and other AI-assisted technologies as an author or co-author and should acknowledge that they are fully responsible for text generated or refined by AI-writing tools.<p><br><br>A summary of use (particularly in the introduction or among methods) needs to be included at the end of the paper, and the outputs should also be included as a supplementary file hosted in GigaDB or other open repositories. Please read our guidelines for more information. <p><br><br>By submitting to GigaScience, you are aware of the journal's AI-writing tools policy, and if you have declared use of such tools below, you have acknowledged this where appropriate in your manuscript and have made a summary of use and outputs available. <p><br><b>AI-assisted writing tools have been	No

used in the preparation of this manuscript?	

Powered by Editorial Manager® and ProduXion Manager® from Aries Systems Corporation

Manuscript

Click here to access/download;Manuscript;TinkerHap-Article-20251027docx.docx

TinkerHap - A Novel Read-Based Phasing Algorithm with Integrated Multi-Method Support for Enhanced Accuracy

Uri Hartmann[1*], Eran Shaham[1], Dafna Nathan[1], Ilana Blech[1], Danny Zeevi[1]

[1]Department of Biotechnology, Jerusalem Multidisciplinary College, Jerusalem, Israel.

*Corresponding author. Email: uri.hartman@edu.jmc.ac.il

ORCID iDs: Uri Hartmann [0009-0002-0668-0249]; Eran Shaham; Dafna Nathan [0000-0003-3338-5579]; Ilana Blech; Danny Zeevi [0000-0002-6901-3737];

Abstract

Phasing, the assignment of alleles to their respective parental chromosomes, is fundamental to studying genetic variation and identifying disease-causing variants. Traditional approaches, including statistical, pedigree-based, and read-based phasing, face challenges such as limited accuracy for rare variants and reliance on external reference panels.

To address these limitations, we developed TinkerHap, a novel phasing algorithm that integrates a read-based phaser, based on a pairwise distance-based unsupervised classification, with external phased data, such as statistical or pedigree phasing. We evaluated TinkerHap's performance against other phasing algorithms using 1,040 parent-offspring trios from the UK Biobank (Illumina short-reads) and GIAB Ashkenazi trio (PacBio long-reads). TinkerHap's read-based phaser alone achieved higher phasing accuracies than all other algorithms with 95.1% for short-reads (second best: 94.8%) and 97.5% for long-reads (second best: 95.5%). Its hybrid approach further enhanced short-read performance to 96.3% accuracy and was able to phase 99.5% of all heterozygous sites. TinkerHap also extended haplotype block sizes to a median of 79,449 base-pairs for long-reads (second best: 68,303 bp) and demonstrated higher accuracy for both SNPs and indels. This combination of a robust

read-based algorithm and hybrid strategy makes TinkerHap a uniquely powerful tool for genomic analyses.

Introduction

Phasing is the process of assigning alleles to their respective maternal or paternal chromosomes. It is essential for determining precise protein sequences in an individual and identifying genes that cause diseases.

Various methods of phasing are available, including statistical phasing based on phased reference genomes (e.g. ShapeIT5 [1] and Beagle [2]), pedigree-based phasing (e.g. LINKPHASE3 [3] and TrioPhaser [4]), and read-based phasing (e.g. WhatsHap [5] and HapCUT2 [6]). Statistical phasing is limited by how well the reference panel represents the sample data and is particularly inaccurate in phasing rare variants [7]. Pedigree-based phasing is accurate for common and rare variants, but pedigree information is typically not available for the inspected individual.

Read-based (or read-aware) phasing works by analyzing sequencing reads that span multiple heterozygous sites to phase them together, resulting in very high accuracy. This method is independent of statistical bias, is unaffected by the rarity of the alleles, and does not require pedigree data, although mapping bias from reference-based alignment remains a known limitation. However, since read-based phasing cannot phase regions where heterozygous sites are farther than read sizes, it can only effectively be used in highly variable regions or when using long-reads that span several heterozygous sites.

Here we present TinkerHap, a read-based phasing tool offering consistent performance and enhanced accuracy. TinkerHap excels in accurately handling rare variants and variable genomic regions, such as the Human Leukocyte Antigen (HLA) locus, while also effectively

phasing long-read data. Moreover, TinkerHap uniquely merges external phasing blocks into its read based framework while maintaining block continuity, which differs from existing tools that do not integrate merging within a read aware propagation step. This hybrid approach bridges gaps in read coverage and extends haplotype blocks.

Methods

Overview

TinkerHap is implemented in Python 3 and utilizes the *pysam* package [8] for manipulating alignment and variant calling files. The command-line interface accepts an alignment file (SAM/BAM/CRAM) and a variant calling file (VCF/BCF) as inputs, producing a phased VCF file through read-based haplotype phasing. Optionally, TinkerHap can integrate a pre-phased VCF file from a third-party tool (e.g., ShapeIT5 [9] for statistical-based phasing) to align and merge haplotypes with greater accuracy when possible.

Additionally, TinkerHap can generate multiple output formats to represent the phased haplotypes: a BED file listing the identified haplotype blocks, a BAM file identical to the original but annotated with haplotype and phase information in the Haplotype Phase field (HP) and Haplotype number field (HT), and two separate BAM files - each containing reads corresponding to one of the phased alleles. These outputs facilitate annotation or the splitting of the original alignment into distinct files for each allele, enabling downstream analyses.

Algorithm

TinkerHap implements a three-step phasing algorithm, based on a pairwise distance-based unsupervised classification, designed for precision. Below is a detailed description of each step with mathematical notations.

1. Identification of Heterozygous Sites

Let $S = \{s_1, s_2, \dots, s_m\}$ represent the set of heterozygous sites identified from the input variant call file (VCF). A site s_i is considered heterozygous if $a_i \neq b_i$ where a_i and b_i are the two alleles at s_i. The loci of these sites are identified as $L(S) = \{l_1, l_2, \dots, l_m\}$, forming the foundation for subsequent phasing steps. Ambiguous allele calls, characterized by low scores in the VCF's QUAL column and typically caused by sequencing or alignment errors, are flagged.

2. Association of Reads with Heterozygous Sites

Let $R = \{r_1, r_2, \dots, r_n\}$ denote the set of sequencing reads. Each read r_j spans a subset of heterozygous sites $S_j \subseteq S$. For each read, we map its alleles to the overlapping sites:

$$A(r_j, s_i) = \begin{cases} a_i, & \text{if allele matches allele 1} \\ b_i, & \text{if allele matches allele 2} \end{cases}$$

This step ensures precise allele identification by linking reads to heterozygous sites while accounting for potential alignment errors or ambiguities, such as indels. To ensure accuracy, only reads meeting a minimum mapping quality threshold (e.g., MAPQ ≥ 20) are considered, ensuring that low-confidence alignments do not influence the phasing process.

3. Calculation of Phase Scores

The phasing process begins by arbitrarily assigning the first read r_1 to one of the haplotypes, for instance H_1. This initial assignment acts as a seed to propagate haplotypes across all overlapping reads.

Each read r is then evaluated to determine its phase matching scores $P_1(r)$ and $P_2(r)$ for the two haplotypes, H_1 and H_2. These scores are computed by analyzing all overlapping reads and the heterozygous sites they share with r.

The phase scores are calculated as:

$$P_H(r) = \sum_{k \in K_r} \sum_{s \in S_r \cap S_k} \Delta P_H(r)$$

Where K_r is the set of all overlapping reads for r, and S_r and S_k are the sets of heterozygous sites for reads r and k, respectively. The contribution of each shared site s to the phase score, $\Delta P_H(r)$, is defined as:

$$\Delta P_H(r) = \begin{cases} +w(s), \text{if } A_r(s) = A_k(s) \text{ and } k \text{ belongs to haplotype } H \\ -w(s), \text{if } A_r(s) \neq A_k(s) \text{ and } k \text{ belongs to haplotype } H \end{cases}$$

Here, $A_r(s)$ and $A_k(s)$ represent the alleles of r and k at site s, respectively. The weight $w(s)$ assigned to site s depends on the type of heterozygous site:

$$w(s) = \begin{cases} 2, \text{if } s \text{ is a SNP} \\ 1, \text{if } s \text{ is an indel} \end{cases}$$

This scoring ensures that the phase scores for r are influenced by the agreement or disagreement between r and all overlapping reads at shared heterozygous sites.

After calculating the phase scores, the haplotype HP of r is assigned as follows:

$$\text{HP}(r) = \begin{cases} H_1, \text{if } P_1(r) > P_2(r), \\ H_2, \text{if } P_2(r) > P_1(r), \end{cases}$$

If $P_1(r)$ and $P_2(r)$ are equal, the haplotype assignment can propagate from the overlapping read with the strongest phase connection, or a new haplotype block may be started. This approach ensures consistency in haplotype assignments based on the majority consensus among overlapping reads.

4. Haplotype Extension

Haplotypes are extended iteratively by analyzing overlapping reads. If a read r_k overlaps two or more phased reads $\{r_{j_1}, r_{j_2}, \ldots\}$, its phase is determined by propagating the majority consensus:

$$\mathrm{HP}(r_k) = \{\mathrm{HP}(r_{j_1}), \mathrm{HP}(r_{j_2}), \ldots\}$$

This ensures the consistency of haplotype assignments across contiguous genomic regions. Reads that span conflicting haplotypes are flagged for manual review or downstream quality filtering.

5. Pair-End Read Merging

For paired-end reads (r_i, r_j), the algorithm evaluates the consistency of their haplotypes:

$$M(r_i, r_j) = \begin{cases} +1, & \text{if } \mathrm{HP}(r_i) = \mathrm{HP}(r_j) \\ -1, & \text{if } \mathrm{HP}(r_i) \neq \mathrm{HP}(r_j) \end{cases}$$

Inconsistent pairs trigger a phase reassignment to minimize discordance, leveraging the paired-end linkage information. A weighted graph representation of pair-end links can be constructed for further optimization of haplotype continuity.

6. Integration with Pre-Phased Data (Optional)

When an additional pre-phased VCF file is provided, for example one generated by statistical phasing tools such as ShapeIT5, the algorithm merges the read-based haplotypes with the pre-phased data. This involves merging haplotypes and, if necessary, switching the phase numbers (e.g., swapping haplotype 1 and haplotype 2) to ensure consistency with the pre-phased data numbering of haplotypes.

The alignment score $A(b, h)$ for a pre-phased block b and a read-based block h is calculated as:

$$A(b,h) = \sum_{s \in S_b \cap S_h} w(s)$$

where S_b and S_h are the sets of heterozygous sites in b and h, and $w(s)$ represents the weight based on the site type (e.g., SNP or indel). Haplotypes are adjusted to maximize $A(b,h)$, ensuring that the merged haplotypes align with the pre-phased data and improving the overall phasing accuracy.

7. Output Generation

The final outputs include:

1. Phased VCF: Annotated with a PS (Phase Set) field.

2. Annotated BAM: Each read is tagged with HP (Haplotype Phase) and HT (Haplotype number) fields.

3. Split BAM Files: Separate BAM files for each haplotype, facilitating downstream analyses.

4. BED File: Haplotype boundaries across the genome are defined for visualization.

The detailed algorithm and code can be accessed at Github. (see Availability of Source Code and Requirements).

Supplementary material for this article, including detailed descriptions of the evaluation and benchmarking procedures, all methodological steps, permissible data, scripts, and resources necessary for reproducibility, is available at Github (see Availability of Source Code and Requirements).

Evaluation

TinkerHap was evaluated in the following use cases:

1. To evaluate the algorithm's performance in variable regions and for rare variants using Illumina short-reads, we analyzed Whole Genome Sequencing (WGS) data from 1,040 parent-offspring trios that we identified in the UK Biobank [10] on the MHC class II region in humans, specifically on chr6:32,439,878-33,143,325 (hg38 genome version).
2. To evaluate the algorithm's performance with long-reads, we used PacBio long-read sequencing data of the full genomes of GIAB Ashkenazi trio HG002-4 and Chinese trio HG005-7 datasets by Revio (publicly offered by GIAB [11]). Results were averaged across the Ashkenazi and Chinese GIAB trios.

For each offspring in the trios, we constructed a "truth" set of known phased heterozygous sites ("truth sites"). This was achieved by examining loci where each parent possesses different homozygous alleles or where one parent was heterozygous, and the other was homozygous. After preparing the data, we phased the offspring sequence using the following algorithms: ShapeIT5[9], WhatsHap[5], HapCUT2[6], TinkerHap, and TinkerHap with ShapeIT5[9] phased data as an additional input for merging haplotypes (as described in the "Algorithm" section above). The success rate was evaluated by counting the number of sites in the phased output that matched the truth set.

All algorithms were run on the same virtual instance type - UK BioBank instance type "mem3_ssd1_v2_x2" (2 cores, 16GB memory, 75GB storage)

Results

MHC class II gene region phasing (chr6:32,439,878-33,143,325)

Criteria	TinkerHap+ ShapeIT5[1]	TinkerHap	WhatsHap	HapCUT2	ShapeIT5
**Phased %**	99.5%	97.1%	86.5%	96.2%	70.5%
**Phasing accuracy %[2]**	96.3%	95.1%	84.9%	94.8%	70.2%

**Phasing accuracy % (SNPs only)**	97.1%	96.0%	86.0%	95.8%	71.6%
**Phasing accuracy % (INDELs only)**	89.6%	87.8%	76.9%	87.4%	59.6%
**Haplotype size (bp)[3]**	21,813	631	75	644	702,123
**Phase blocks[4]**	11.5	82	325	84	1
**Phase blocks N50[5] (bp)**	139,281	19,596	1,489	13,678	702,123
**Total phased size (bp)**	623,829	329,037	119,554	276,724	702123
**Common phased sites errors[6]**	0.14%	0.14%	0.18%	0.12%	0.17%
**Coverage**	x32.5				
**Heterozygous sites[7]**	5,086				
**Runtime (s)[8]**	6.8	6.5	23.8	9.2	13.7
**Bases / second**	104,005	108,245	29,532	76,105	51,545
**Heterozygous sites / second**	743	773	211	544	368
**Memory usage (MB)**	109	106	397	594	24

Table 1. **Phasing performance of different algorithms on short-reads aligned to the MHC class II region**

[1] TinkerHap+ShapeIT5: TinkerHap algorithm when used with additional ShapeIT5 pre-phased file.
[2] Phasing accuracy: Successfully phased sites divided by the total number of heterozygous sites.
[3] Haplotype size: Median haplotype size across all samples. "Haplotype" refers to a set of alleles at variant sites along a single chromosome that are inherited together and are guaranteed to be phased together by the algorithm.
[4] Phase blocks: Median number of phase blocks across all samples.
[5] Phase blocks N50: median of the N50 haplotype block sizes computed individually for each sample, providing a summary measure of typical haplotype block contiguity across the dataset.
[6] Common phased sites errors: Phasing error % in heterozygous sites phased by all algorithms.
[7] Heterozygous sites: Median number of heterozygous sites across all samples.
[8] Runtime: Median runtime per sample.

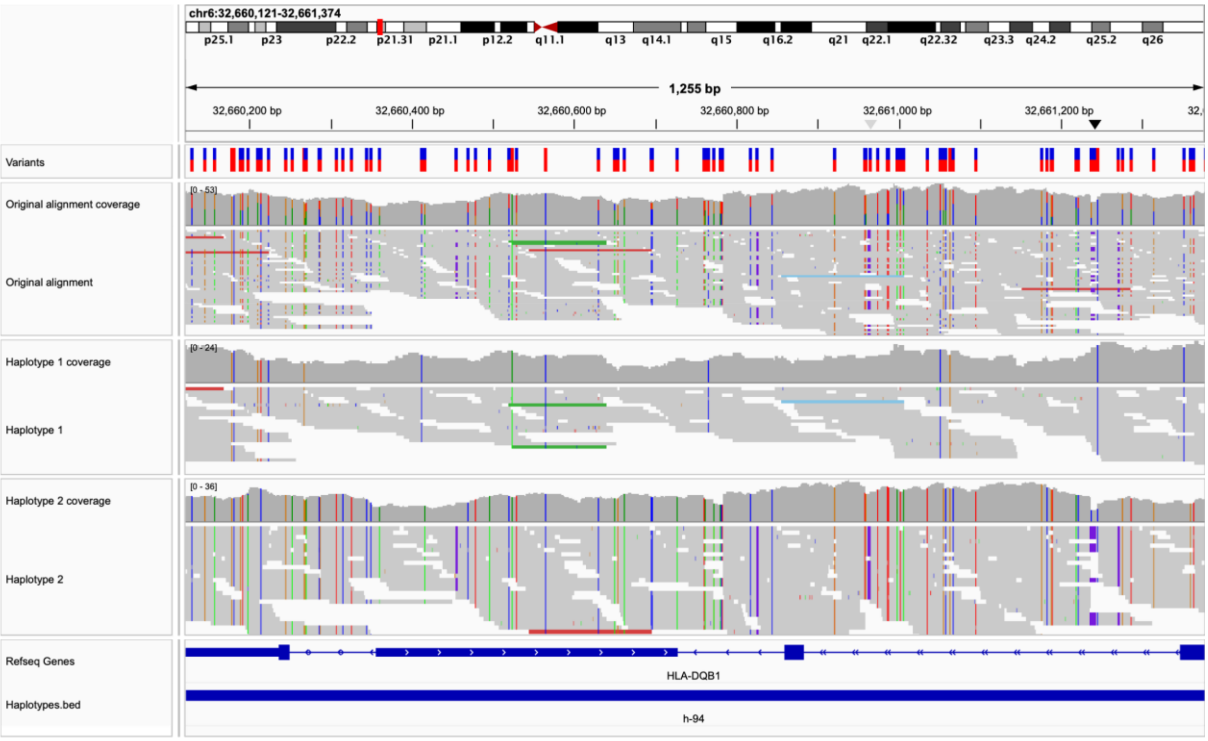

Figure 1. Phased BAM Outputs displaying Heterozygous sites (IGV [12] screenshot) - The upper track is the original alignment, while the two tracks below represent the output of Haplotype 1 and Haplotype 2. Heterozygous sites are correctly segregated between the two phases, demonstrating successful phasing. The continuous blue line in the bottom track illustrates a BED file annotation, highlighting the size of the haplotype region where all variants are confirmed to share the same phase.

PacBio phasing (whole genome)

Criteria	TinkerHap+ ShapeIT5	TinkerHap	WhatsHap	HapCUT2	ShapeIT5
Phased %	99.8%	99.4%	96.8%	96.7%	87.84%
Phasing accuracy %[1]	97.8%	97.5%	95.5%	95.4%	86.68%
Phasing accuracy % (SNPs only)	98.0%	97.8%	96.5%	96.4%	89.0%
Phasing accuracy % (INDELs only)	96.35%	96.0%	90.2%	90.5%	75.25%
Haplotype size (bp)[2]	809,769	79,449	68,303	72,220	133,491,606
Phase blocks[3]	1,490	10,061	10,561	10,586	22
Phase blocks N50[4] (bp)	4,603,981	614,204	558,942	558,942	133,491,606
Total phased size (Mbp)	2,675	2,275	2,173	2,171	2,807

**Common phased sites errors[5]**	0.14%	0.14%	0.15%	0.20%	0.60%
**Coverage**	x50				
**Heterozygous sites**	100,910				
**Runtime (s)[6]**	10,797	10,519	17,578	5,495	27,474
**Bases / second**	290,572	298,239	178,475	570,891	114,191
**Heterozygous sites / second**	421	433	259	829	166
**Memory usage (MB)**	1,780	1,726	1,696	876	2,189

Table 2. Phasing performance of different algorithms on long-reads of full genomes.
[1] Phasing accuracy: Successfully phased sites divided by the total number of heterozygous sites.
[2] Haplotype size: Median haplotype size across all samples.
[3] Phase blocks: Average number of phase blocks across all samples.
[4] Phase blocks N50: median of the N50 haplotype block sizes computed
[5] Common phased sites errors: Phasing error % in heterozygous sites phased by all algorithms.
[6] Runtime: Average runtime per sample.

Discussion

Here, we introduce TinkerHap, a read-based phasing algorithm designed for accurate and reliable phasing across diverse genomic contexts, with the ability to integrate statistical phasing data from third-party tools for improved performance.

We evaluated TinkerHap using two datasets: the MHC class II region in humans with Illumina short-read WGS data to assess its accuracy in variable regions, and PacBio sequencing data to evaluate its performance with long-reads. These datasets were selected due to their suitability for testing read-based phasing algorithms, as both are characterized by a high density of variants that provide many opportunities for phasing.

Performance of Short-Reads in Variable Regions

In the MHC class II region using short-reads, TinkerHap phased 97.1% of variants with 95.1% accuracy. In comparison, the second-best algorithm phased 96.2% of variants with

94.8% accuracy. All methods showed higher phasing accuracy for SNPs compared to indels (97.1% and 89.6%, respectively, in TinkerHap).

Performance of Long-Read Sequencing

TinkerHap achieved a phasing accuracy of 97.5% for SNPs and 96.0% for indels with PacBio datasets. These results were superior to the second-best algorithm, which demonstrated accuracies of 95.5% and 95.4%, respectively. Moreover, TinkerHap produced longer haplotype blocks (median size: 79,449 bp) compared to the second-best algorithm (68,303 bp). Runtime analysis revealed that TinkerHap required 10,519 seconds per sample, compared to 5,495 seconds for the fastest algorithm.

Comparison of Long-Read and Short-Read Performance

An important advantage of TinkerHap, as well as all read-based algorithms, is that it is less likely to be affected by the rarity of a variant [7]. However, the density of the variants in combination with the length of the sequencing read are key factors for the performance of TinkerHap. A key limitation of read-based phasing approaches, including TinkerHap, arises from the typical length of short sequencing reads (~150 bp) relative to the spacing between variants (500–1,000 bp on average). As a result, individual reads seldom span multiple variants, thereby yielding little to no additional or informative phasing data. This limitation confines the effective application of read-based phasing methods like TinkerHap primarily to long-read sequencing projects, where reads are sufficiently extended to span multiple variants, or to genomic regions exhibiting high variant density (greater than approximately 1 variant per 100 bp), such as the human leukocyte antigen (HLA) locus.

Accordingly, TinkerHap performed better with long-read sequencing data compared to short-read data in several key metrics. Long-reads offer superior upstream alignment quality, particularly at highly variable sites, which enhances the overall accuracy of variant calling

and subsequent phasing steps. Long-read data yielded more extensive haplotype blocks (median size: 79,449 bp compared to 631 bp with short-reads) and higher phasing accuracy (97.5% for SNPs in long-reads compared to 96.0% in short-reads, and 96% for indels in long-reads compared to 87.8% in short-reads). This improved performance is expected due to long-reads containing more heterozygote sites and enabling improved alignments.

Integration with Statistical Phasing

TinkerHap uniquely includes the ability to integrate data from third-party tools, such as ShapeIT5. By incorporating pre-phased haplotypes, the TinkerHap + ShapeIT5 combination achieved 99.5% phased variants with 96.3% accuracy, significantly outperforming standalone methods. This hybrid approach improved haplotype block continuity and effectively addressed gaps in read coverage.

Limitations

TinkerHap's runtime and memory usage for long-read data present areas for potential optimization, and it currently lacks support for polyploid genomes. TinkerHap is limited in merging distant haplotypes, which could be particularly useful for applications such as exome sequencing. Future incorporation of pedigree information could address this issue and enhance TinkerHap's accuracy in trio or family-based studies.

In most phasing errors that we manually examined, inaccuracies were primarily attributed to upstream variant calling rather than to the phasing algorithm itself. This suggests that TinkerHap may be approaching the limit of what can be achieved with downstream read-based phasing alone. This underscores the importance of high-quality preprocessing.

Availability of supporting source code and requirements

Project name: TinkerHap

Project home page: https://github.com/DZeevi-Lab/TinkerHap

Supplementary information: https://github.com/DZeevi-Lab/TinkerHap-Supplementary

Operating system(s): Platform independent

Programming language: Python

Other requirements: Python 3.6.0 or higher, pysam 0.17 or higher

License: MIT

RRID: SCR_027235

bio.tools ID: tinkerhap

Data Availability

UK Biobank data: The Illumina short-read data from 1,040 parent-offspring trios used in this study were accessed from the UK Biobank under application number 74655. These data are available under controlled access due to participant privacy considerations. Researchers can apply for access through the UK Biobank Access Management System by submitting a detailed research proposal. Further information and application guidelines are available at [13].

Genome in a Bottle (GIAB) data: The long-read sequencing data from the GIAB Ashkenazi and Chinese trios used for algorithm evaluation are publicly available at the National Center for Biotechnology Information (NCBI) FTP site. The datasets can be accessed directly via ftp [14]. Further information is available in the supplementary material for this article.

Funding

This research was supported by the ISRAEL SCIENCE FOUNDATION and JDRF (grant No. 2658/21).

Competing interests

The authors declare no competing interests.

Author contribution:

Conceptualization – U.H., D.Z.; Methodology - U.H.; Formal Analysis - U.H.; Investigation - U.H; Writing, original draft preparation – U.H.; Writing, review & editing - U.H., D.Z., E.S., D.N, I.B.; Visualization - U.H. Supervision – D.Z.; Funding Acquisition – D.Z.

Acknowledgements:

We thank Rona Gershon Talmi from the Hamaabada Podcast (Kan) and Dr. Jeremy Fogel and Tuval Rosenwasser from the Think & Drink Different Podcast for their contribution to this work.

This research has been conducted using the UK Biobank Resource under application number 74655.

References

[1] R. J. Hofmeister, D. M. Ribeiro, S. Rubinacci, and O. Delaneau, "Accurate rare variant phasing of whole-genome and whole-exome sequencing data in the UK Biobank," *Nat Genet*, vol. 55, no. 7, 2023, doi: 10.1038/s41588-023-01415-w.

[2] B. L. Browning, X. Tian, Y. Zhou, and S. R. Browning, "Fast two-stage phasing of large-scale sequence data," *The American Journal of Human Genetics*, vol. 108, no. 10, pp. 1880–1890, Oct. 2021, doi: 10.1016/j.ajhg.2021.08.005.

[3] T. Druet and M. Georges, "LINKPHASE3: an improved pedigree-based phasing algorithm robust to genotyping and map errors," *Bioinformatics*, vol. 31, no. 10, pp. 1677–1679, May 2015, doi: 10.1093/bioinformatics/btu859.

[4] D. B. Miller and S. R. Piccolo, "trioPhaser: using Mendelian inheritance logic to improve genomic phasing of trios," *BMC Bioinformatics*, vol. 22, no. 1, p. 559, Dec. 2021, doi: 10.1186/s12859-021-04470-4.

[5] M. Martin *et al.*, "WhatsHap: fast and accurate read-based phasing," *bioRxiv*, p. 085050, Nov. 2016, doi: 10.1101/085050.

[6] V. Bansal, "HapCUT2: A Method for Phasing Genomes Using Experimental Sequence Data," *Methods in Molecular Biology*, vol. 2590, pp. 139–147, 2023, doi: 10.1007/978-1-0716-2819-5_9.

[7] Y. Choi, A. P. Chan, E. Kirkness, A. Telenti, and N. J. Schork, "Comparison of phasing strategies for whole human genomes," *PLoS Genet*, vol. 14, no. 4, p. e1007308, Apr. 2018, doi: 10.1371/journal.pgen.1007308.

[8] A. Heger, "Pysam: HTSlib interface for Python, 2009," https://github.com/pysam-developers/pysam.

[9] O. Delaneau, J. Marchini, and J. F. Zagury, "A linear complexity phasing method for thousands of genomes," *Nature Methods 2011 9:2*, vol. 9, no. 2, pp. 179–181, Dec. 2011, doi: 10.1038/nmeth.1785.

[10] C. Sudlow *et al.*, "UK Biobank: An Open Access Resource for Identifying the Causes of a Wide Range of Complex Diseases of Middle and Old Age," *PLoS Med*, vol. 12, no. 3, p. 1001779, Mar. 2015, doi: 10.1371/JOURNAL.PMED.1001779.

[11] Genome in a Bottle Consortium, "GIAB Benchmarking Data," 2023, Accessed: Dec. 12, 2024. [Online]. Available: https://ftp-trace.ncbi.nlm.nih.gov/ReferenceSamples/giab/data/

[12] H. Thorvaldsdottir, J. T. Robinson, and J. P. Mesirov, "Integrative Genomics Viewer (IGV): high-performance genomics data visualization and exploration," *Brief Bioinform*, vol. 14, no. 2, pp. 178–192, Mar. 2013, doi: 10.1093/bib/bbs017.

[13] Application guidelines for UK Biobank data https://www.ukbiobank.ac.uk/enable-your-research/apply-for-access.

[14] FTP for Genome in a Bottle (GIAB) data: https://ftp-trace.ncbi.nlm.nih.gov/ReferenceSamples/giab/data/

Click here to access/download
Supplementary Material
TinkerHap-Supplementary-202509.docx

Personal Cover Click here to access/download;Personal Cover;personal-cover.docx

May 18, 2025

Dear Editor,

I am writing on behalf of my coauthors to submit our manuscript, “TinkerHap – A Novel Read-Based Phasing Algorithm with Integrated Multi-Method Support for Enhanced Accuracy,” for consideration as a Technical Note in GigaScience.

In this work we developed a novel and unique algorithm for phasing. Phasing is the assignment of alleles to their respective parental chromosomes. It is crucial for our understanding of how certain allelic combinations drive disease risk, especially in complex traits.

Various methods of phasing are currently available, but each suffers significant inaccuracies and challenges when applied alone. Statistical phasing methods are limited by how well a reference panel represents the sample data and are particularly inaccurate in phasing rare variants (which are the most important variants for identifying the genetic basis of diseases). Pedigree-based phasing is typically not available for an inspected individual, and read-based phasing is limited in regions with sparse genetic variation (especially coding regions) and produces short haplotype blocks.

Our method goes beyond traditional phasing approaches by integrating different methods together in several steps.

For the first step we developed a new read-based phaser, based on a pairwise distance-based unsupervised classification. We then combined our read-based algorithm with externally phased data, such as statistical or pedigree phasing, to improve the accuracy of the phasing and increase the length of the haplotype blocks. This hybrid approach resulted in significant improvement of phasing over stand-alone algorithms. We tested TinkerHap hybrid algorithm and three other algorithms (WhatsHap, HapCUT2 and ShapeIT) on UK Biobank trios on the MHC class 2 genomic region that was sequenced with Illumina short reads. Hybrid TinkerHap was able to phase 99.5% of heterozygote sites, with 96.3% accuracy and 21,831 bps mean haplotype block length. HapCUT2, the second best algorithm, phased 96.2% of heterozygote sites, at 94.8% accuracy and with mean haplotype block size of only 644 bps. ShapeIT, which is a stand-alone statistical based phaser was able to phase only 70.5% of the heterozygote sites.

As the median gene size in the human genome is approximately 24,000 bps, TinkerHap is the first phasing algorithm that can phase entire genes with extremely high accuracy. This is a crucial advancement for studies of the genetic basis of traits and diseases, as it allows to accurately infer the two full human protein products of a gene from DNA sequencing.

Lastly, the recent shift to whole genome sequencing (WGS) in biobanks (e.g. the UK Biobank published the entire cohort 500,000 whole genomes approximately a year ago) offers an unprecedented opportunity for large-scale phasing that can serve multiple researchers in studying

multiple diseases and traits. An accurate phasing algorithm with the ability to phase large haplotype blocks will enable researchers to uncover complex patterns of inheritance and identify how groups of phased variants interact to cause complex diseases.

All authors have approved this manuscript submission for publication. There are no conflicts of interest to declare. We are grateful for your consideration of our work and would welcome the opportunity to provide any additional information that might assist the review process.

Thank you for your time and consideration. We look forward to your response.

Sincerely,

Danny Zeevi, PhD

Chair, Department of Biotechnology

Jerusalem Multidisciplinary College, Jerusalem, Israel

We would like to thank you and the reviewers for the constructive and thoughtful peer review of our manuscript. We are hereby submitting the revised version of the manuscript along with the supplementary material. In addition, we have uploaded to the SFTP server the relevant files associated with the additions of TinkerHap+ShapeIT and ShapeIT, and we have also updated the TinkerHap-Supplementary repository on GitHub.

Email points:

One important topic mentioned in the reports is the need to present an improved and fair assessment of the method, also with a wider scope, e.g. on a chromosome-/ genome-wide scale.
We agree that this is a very important point. Therefore, in the second part of the analysis we included a genome-wide evaluation of our method on samples from the GIAB project, which provides a comprehensive benchmark across the full genome. It is important to note that such analysis is relevant only when using long reads. Since short sequencing reads are typically ~150 bp long, and variants are typically 500-1000 bp apart, individual sequencing reads rarely cover more than one variant, so any read-based phasing method such as TinkerHap would not produce additional or meaningful phasing information. This is why we focused on our short-read analysis on the MHC class II region which is the most variable in the genome.

To emphasize these limitations of our method and make it clearer to the readers, we added the following paragraph to the discussion section:

“A key limitation of read-based phasing approaches, including TinkerHap, arises from the typical length of short sequencing reads (~150 bp) relative to the spacing between variants (500–1,000 bp on average). As a result, individual reads seldom span multiple variants, thereby yielding little to no additional or informative phasing data. This limitation confines the effective application of read-based phasing methods like TinkerHap primarily to long-read sequencing projects, where reads are sufficiently extended to span multiple variants, or to genomic regions exhibiting high variant density (greater than approximately 1 variant per 100 bp), such as the human leukocyte antigen (HLA) locus.”

In addition to addressing the reviewers' comments, please register any new software application in the bio.tools and SciCrunch.org databases to receive RRID (Research Resource Identification Initiative ID) and biotoolsID identifiers, and include these in your manuscript. Computational workflows should be registered in workflowhub.eu and the DOIs cited in the relevant places in the manuscript. These will facilitate tracking, reproducibility and re-use of your tool.
We thank you and the reviewers for pointing out these important databases. We have registered TinkerHap in the bio.tools and SciCrunch.org databases, and the manuscript now includes the assigned biotoolsID and RRID identifiers to facilitate tracking, reproducibility, and reuse of the tool. Regarding WorkflowHub.eu, we believe this registry is less applicable to TinkerHap, as TinkerHap is a standalone software tool for read-based variant phasing rather than a multi-step computational workflow.

Reviewer reports:

Reviewer #1: The authors present TinkerHap, a tool that accepts a variant call set and read alignment, and assigns heterozygous variants and reads to a particular haplotype based on

a greedy pairwise distance-based classification. It accepts a pre-phased VCF as an option to further extend phased blocks.
The results sound neat with statistics making it look the greatest compared to current state-of-the-art read alignment based phasing methods such as HapCut2, WhatsHap, and ShapeIT which uses statistical inference from reference panel data. However, there are several aspects the authors need to address to make their results more compelling.

1. The benchmarking was only performed on MHC Class II, which is a relatively small and easy to phase region based on the high level of heterozygosity. How does the statistics look when applied to the whole genome? After generating the phased read set, what % of reads can be accurately assigned to the original haplotype in the whole genome scale? To benchmark the latter, I would recommend doing it on HG002 phased variants and reads by using the HG002Q100 genome (https://github.com/marbl/hg002) - i.e. map the classified reads and calculate the coverage and accuracy based on where the reads align to. I would be curious to see how the MHC Class II phased read alignment looks like on the HG002Q100 truth assembly, on each haplotype.

   Thank you very much for your thoughtful comments and suggestions.

   Regarding the benchmarking on MHC Class II, we acknowledge that this region benefits from high heterozygosity, making it particularly suitable for phasing. We agree that it is essential to test TinkerHap on a genome-wide scale. Therefore, we have already analyzed TinkerHap's performance on the whole-genome scale for HG002 and an additional GIAB sample, with the results presented in Table 2.

   For short-reads, since short sequencing reads are typically ~150 bp long, and variants are typically 500-1000 bp apart, individual sequencing reads rarely cover more than one variant, so applying any read-based phasing method such as TinkerHap on short reads would not produce additional or meaningful phasing information to many regions of the genome. To address this, we have now added a caveat to the Discussion section highlighting this limitation of read-based phasing methods like TinkerHap:

   "A key limitation of read-based phasing approaches, including TinkerHap, arises from the typical length of short sequencing reads (~150 bp) relative to the spacing between variants (500–1,000 bp on average). As a result, individual reads seldom span multiple variants, thereby yielding little to no additional or informative phasing data. This limitation confines the effective application of read-based phasing methods like TinkerHap primarily to long-read sequencing projects, where reads are sufficiently extended to span multiple variants, or to genomic regions exhibiting high variant density (greater than approximately 1 variant per 100 bp), such as the human leukocyte antigen (HLA) locus."

   Regarding the MHC Class II phased read alignment performance on truth assembly such as HG002Q100, it is important to note that we benchmarked TinkerHap on approximately 1,000 diverse short-read samples of trios, with truth phasing learned from parent-offspring inheritance.

2. When showing benchmarking results, key features are missing - 1) number of heterozygous variant sites are used for phasing, in addition to the Phased % (what's the denominator here?), 2) number of phase blocks, phase block NG50 and total

length and 3) Show the NGx length distribution by plotting the cumulative covered genome length as a function of the longest to shortest phase block.
We agree, and added the number of heterozygous variant sites used for phasing to the manuscript as well as the number of phase blocks, phase block N50 and the total length of the phased blocks.

3. After phasing the variants (and reads), are the authors accurately able to type the HLA Class II genes? The goal of MHC phasing is to accurately genotype the HLA-genes. It is unclear to me why the authors applied their phasing on the 1,040 parent-offspring trios. I agree that it is 'phasable', however, it is unclear what the motivation here is - the MHC Class II is particularly known to have linked HLA types (e.g., HLA-DRB3 and HLA-DRB5 are inherited together depending on the HLA-DRB1 type, while in some haplotypes HLA-DRB3 is entirely missing), and depending on the HLA types and because the reference is incompletely representing this locus, there are multiple tools developed for genotyping this locus. I would be more convinced if the authors could show the HLA genotyping accuracy together based on their phasing method.
We agree that accurate typing of HLA Class II genes is very important. In this work we chose to evaluate phasing performance based on truth datasets learned from trios, which is the golden standard for phasing evaluation. Testing the accuracy of phasing based on the accuracy of typing might introduce biases due to the different methods the reference typing sets were created by.
4. Is it possible to use additional data types to further extend the phase blocks, by using datasets such as low coverage PacBio data in addition to the short-read WGS? How about phasing with linked-reads or Hi-C? Both Whatshap and HapCut2 are specifically designed to combine such short and long-range datasets, giving the advantage of using such tools.
We agree that long reads such as PacBio and other techniques such as Hi-C can contribute to evaluating TinkerHap performance and to extend phasing blocks. Therefore, we have analyzed TinkerHap's performance on PacBio long reads and the results are presented in Table 2. We did not have access to Hi-C linked reads in combination with its raw data sequencing reads, and we believe that such broader comparison that integrates multiple additional technologies and tools would be better suited to a larger dedicated study, and we view this as an interesting direction for future work.
5. The authors claim their method is free from reference bias, which I strongly disagree. Using a bam file aligned to a reference inherently has the issue of mapping biases, so any such tools are limited by the reads that aligns incorrectly. Repeats, especially copy number variable region with collapses in the reference are very difficult to accurately phase. Any large structural variant not properly represented in the reference will cause problems due to unmapped reads.
Very good point, the differentiation between reference bias in multiple phased genomes and reference bias of the reference genome was not clear in our manuscript. The manuscript has been revised to explain that the method itself is free from statistical bias, however mapping bias from reference-based alignment remains a known limitation
6. In Methods, 2nd section - I would suggest to use allele 1 and allele 2 instead of 'reference' and 'alternative' in the equation and the code. This will increase the number of heterozygous 'phasable' variants that does not carry any reference allele.
We thank the reviewer for this excellent suggestion. The manuscript and corresponding code have been updated to use "allele 1" and "allele 2" instead of "reference" and "alternative" in the relevant section.

Reviewer #2: TinkerHap is a read-based phasing algorithm designed to accurately assign alleles to parental haplotypes using sequencing reads.
General comments:
The manuscript would greatly benefit from the inclusion of a flowchart or schematic overview of the TinkerHap algorithm. Given that the method incorporates multiple components—including read-based phasing, pairwise distance-based unsupervised classification, and optional integration with statistical phasing tools like ShapeIT—a visual diagram would help readers grasp the workflow more intuitively.
Major comments:

1. The authors are missing experiments for long-read based phasing. How does TinkerHap performs with ShapeIT on PacBio long-reads? I would suggest the authors using the same phasing method class as their short-read analysis: TinkerHap+ShapeIT; TinkerHap; WhatsHap; HapCUT2; ShapeIT. Also I believe ShapeIT is capable to take long-read SNV/INDEL calls as vcf.
We thank the reviewer for this valuable comment and fully agree with the suggestion. In response, we have extended our PacBio long-read benchmarking to include both TinkerHap+ShapeIT and ShapeIT alone, alongside the other methods. These additions provide a more complete and balanced evaluation of the different phasing strategies, and the results are now included in Table 2 of the revised manuscript.
2. Following up on the point 1, the experimental design of this study is quite skewed. WhatsHap is not suitable for short-read sequencing data. It does not make sense to apply WhatsHap on short-read data.
This is a very good point. We compared WhatsHap on PacBio long reads (table 2), and used it for short reads only on the MHC class II region, because this region contains sufficient variant density for effective read-based phasing even with short-reads, making it comparable in applicability to TinkerHap. We have now also added the number of heterozygous variant sites used for phasing, which allows the reader a better understanding of the framework that the different methods were compared in
3. I would caution the authors to read and potentially compare with SAPPHIRE (https://doi.org/10.1371/journal.pgen.1011092). This is a method that developed by the ShapeIT team for incorporating long-read sequencing data and ShapeIT.
SAPPHIRE is a great tool for phasing, that includes ancestry-aware phasing of large-scale genotype datasets. However it does not accept a .bam or .cram alignment files as input, and does not include a read-based feature. In this work we focused on benchmarking against well-established and widely adopted read-based phasing methods to provide a stable and recognized reference frame. Combining TinkerHap with additional categories of phasing tools in order to get better phasing is a great option for future work.
To better justify the hybrid strategy, I recommend adding an analysis of sites where TinkerHap and ShapeIT disagree. Are these differences due to reference bias, read coverage, variant type, or true ambiguity? Such an evaluation would help users understand when to rely on the read-based output vs. ShapeIT, and enhance confidence in the merging strategy.
An in-depth disagreement analysis should be very interesting and insightful. Since in many cases the determination of the reason for discrepancy might be impossible to do with certainty and can cause bias in the analysis, the major difference between ShapeIT and TinkerHap is that ShapeIT (and other statistical phasing algorithms) is less likely to accurately phase rare variants, while TinkerHap (and other read-based algorithms) are not affected by the frequency of the variant, but rather by the density of the variants and length of reads. For example, a previous study has shown that for

ShapeIT, the switch error rate (SER) increases as the minor allele frequency (MAF) decreases, particularly for MAF below 1% (Choi et al., 2018).
To make it clearer to the readers, we have added to the manuscript the following paragraph in the discussion:
“An important advantage of TinkerHap, as well as all read-based algorithms, is that it is less likely to be affected by the rarity of a variant (Choi et al., 2018). However, the density of the variants in combination with the length of the sequencing read are key factors for the performance of TinkerHap. A key limitation of read-based phasing approaches, including TinkerHap, arises from the typical length of short sequencing reads (~150 bp) relative to the spacing between variants (500–1,000 bp on average). As a result, individual reads seldom span multiple variants, thereby yielding little to no additional or informative phasing data. This limitation confines the effective application of read-based phasing methods like TinkerHap primarily to long-read sequencing projects, where reads are sufficiently extended to span multiple variants, or to genomic regions exhibiting high variant density (greater than approximately 1 variant per 100 bp), such as the human leukocyte antigen (HLA) locus.”

Minor comments:

1. I could see the versions of the software in the supplementary github, but I think it is also important to include those in the manuscript. For example, shapeIT 2-5 are having quite different functions. The citation for ShapeIT in the manuscript is for ShapeIT 2, but the program that has been used is for ShapeIT 5.
   Thank you for noticing this. The manuscript has been revised so that the citation now correctly matches the version of ShapeIT that was used.
2. Need to mention the benchmarking hardware information for runtime comparison.
   Thank you for pointing this out. The benchmarking hardware information was already included in the supplementary material, but we now added it also to the main manuscript.
3. "...a novel and unique phasing algorithm..." -> "...a novel phasing algorithm..."
   Thank you for ponting this out - The wording has been updated accordingly in the manuscript.

Reviewer #3: In the presented Technical Note "TinkerHap - A Novel Read-Based Phasing Algorithm with Integrated Multi-Method Support for Enhanced Accuracy" by Hartmann et al., the authors introduce TinkerHap, a new hybrid phasing tool that primarily relies on read-based phasing for both short- and long-read sequencing data, but can additionally incorporate externally phased haplotypes, enabling it to build upon phase information derived from existing statistical or pedigree-based phasing approaches. This hybrid approach addresses an important and timely challenge in the field: integrating the complementary strengths of different phasing strategies to improve the accuracy and span of haplotype blocks, particularly for rare variants, or in variant-sparse genomic regions. The authors clearly articulate the limitations of existing approaches and present their solution in a manner that is both elegant and accessible. Design features such as multiple output formats and
compatibility with third-party tools demonstrate a practical awareness of user needs. The authors evaluate TinkerHap using both short-read and long-read state-of-the-art benchmarking datasets, and compare its performance against commonly used phasing tools, demonstrating improvements in both phasing accuracy and haplotype block lengths. Overall, this is a well-conceived and thoughtfully implemented contribution to the phasing community.

While the manuscript is overall well written, there are a few areas where additional clarification or extension would improve its impact. I recommend the following revisions to help clarify key aspects of the method, enhance the generalizability of the evaluation, and align the manuscript more closely with journal guidelines.

Major Comments:

1. Limited scope of benchmarking
   The evaluation on the highly polymorphic MHC class II region is appropriate for highlighting TinkerHap's strengths in phasing rare variants in variable regions. However, the current evaluation on short -read based phasing is based on a ~700 kb region selected for its high variant density, which limits the generalizability of the findings. Since the manuscript emphasizes improved performance in regions with sparse genetic variation, it would strengthen the work to include chromosome-wide or genome-wide benchmarks, particularly on short-read data. This would also provide a more balanced comparison with tools like SHAPEIT5, which predictably underperform in the MHC class II region due to their reliance on population allele frequencies and linkage disequilibrium patterns that are less effective for rare or private variants.
   Thank you very much for your thoughtful comments and suggestions.

   Regarding the benchmarking on MHC Class II, we acknowledge that this region benefits from high heterozygosity, making it particularly suitable for phasing. We agree that it is essential to test TinkerHap on a genome-wide scale. Therefore, we have already analyzed TinkerHap's performance on the whole-genome scale on GIAB samples, with the results presented in Table 2.

   For short-reads, since short sequencing reads are typically ~150 bp long, and variants are typically 500-1000 bp apart on average in the genome, individual sequencing reads rarely cover more than one variant, so applying any read-based phasing method such as TinkerHap on short reads would not produce additional or meaningful phasing information to most regions of the genome. To address this, we have now added a caveat to the Discussion section highlighting this limitation of read-based phasing methods like TinkerHap:

   “A key limitation of read-based phasing approaches, including TinkerHap, arises from the typical length of short sequencing reads (~150 bp) relative to the spacing between variants (500–1,000 bp on average). As a result, individual reads seldom span multiple variants, thereby yielding little to no additional or informative phasing data. This limitation confines the effective application of read-based phasing methods like TinkerHap primarily to long-read sequencing projects, where reads are sufficiently extended to span multiple variants, or to genomic regions exhibiting high variant density (greater than approximately 1 variant per 100 bp), such as the human leukocyte antigen (HLA) locus.”
   We have also revised the text to remove the reference to “sparse genetic variation”.

2. Coverage and scalability
   The manuscript describes TinkerHap as scalable, but since the algorithm relies on overlapping reads, it is unclear how its performance varies with sequencing depth. Including a figure or supplementary analysis showing phasing accuracy, runtime, and memory usage at different coverage levels (particularly for short-read data) would help support this claim and guide users on appropriate coverage requirements.
   Thank you pointing this out. The manuscript includes comparisons of runtime and

memory usage, which offer practical insights into the tool's scalability relative to other methods. To prevent any overstatement, we have removed the phrase "and scalability" from the algorithm description.

3. Clarify algorithmic novelty

   It would be helpful to elaborate on how TinkerHap's read-based phasing algorithm differs from existing approaches such as the weighted Minimum Error Correction (wMEC) framework implemented in WhatsHap. For example, what specifically enables TinkerHap's read-based mode to produce longer haplotype blocks than other read-based tools?

   The key difference between TinkerHap and existing approaches such as WhatsHap lies in the algorithmic strategy used to address this NP-hard problem. WhatsHap's runtime complexity is O(2k m), where k is the maximum read coverage at any variant site, and m is the number of SNP positions. To avoid exponential runtime, WhatsHap enforces an internal coverage cap (flag: "--internal-downsampling"), with a default k=15 and a hard limit of k=23. As shown in Figure 2 of the WhatsHap paper, reducing coverage increases both the error rate and the proportion of unphasable positions.

   In our experiments, we used data from the UK BioBank. This data has an average coverage of ×32.5. At this depth, WhatsHap would either (i) run with an unfeasible runtime if coverage is not capped, or (ii) require downsampling, leading to higher error rates and more unphasable positions.

   In contrast, TinkerHap formulates read-based phasing as a K=2 clustering problem. It takes an iterative greedy expansion approach, with an overall polynomial runtime. Like other clustering algorithms (e.g., K-Means), TinkerHap is sensitive to initialization. However, this can be mitigated with multiple runs with different initializations.

   To conclude, TinkerHap enjoys a polynomial runtime, while harnessing the full benefits of a high coverage to produce longer haplotype blocks with fewer errors and unphasable sites.

4. Data description

   A brief characterization of the input datasets, such as the sequencing depth, as well as the number and average genomic distance of heterozygous variants in the MHC class II region and the GIAB trio data would provide important context for interpreting the reported phasing accuracy and haplotype block lengths.

   - We agree it is very important, and so sequencing depth and the number of heterozygous variant sites used for phasing have now been added to the manuscript.

5. Manuscript structure

   Since the algorithm itself is the core novel contribution, it should be part of the results section, as well as the description of the evaluation currently in placed in the discussion. According to GigaScience's Technical Note guidelines, the method section should be reserved for "any additional methods used in the manuscript, that are not part of the new work being described in the manuscript."

   Thank you very much for your thoughtful suggestion regarding the manuscript's structure and for referencing GigaScience's Technical Note guidelines. We greatly appreciate your attention to detail in ensuring alignment with journal standards.

   Upon careful review of the guidelines, we recognize that the Methods section is intended for additional methods not part of the new work, while the Findings section should detail the implementation, availability, and testing of the novel contribution in a reproducible manner. In our manuscript, we placed the TinkerHap algorithm description in Methods to provide a dedicated space for the technical details needed for reproduction, as this aligns with another requirement of the technical guidelines for the method section - "This section should provide enough detail to allow other

researchers to interpret and repeat the study.” We also think that conventional expectations in bioinformatics papers are that researchers often look to Methods for algorithmic specifics to interpret and repeat the work, while focusing on the results section for evaluation and benchmarking.
To better adhere to the guidelines without major restructuring, we have added cross-references and a subsection in Results explicitly linking to the algorithm's implementation details, emphasizing reproducibility. ~Based on the algorithm described in the Methods section...~

Minor Comments

1. Novelty of hybrid approach
   While TinkerHap's ability to integrate externally phased haplotypes is valuable, similar functionality exists in other tools, for example, SHAPEIT can accept pre-phased scaffolds (including those generated from read-based phasing), and WhatsHap supports trio-based phasing. Consider refining the language to more precisely describe what is uniquely implemented in TinkerHap's hybrid strategy. It would be interesting to see how the presented results of using SHAPEIT's phasing output as input for TinkerHap compare to an approach of feeding TinkerHap's read-based phasing results into SHAPEIT.
   Great point. The introduction text now specifies that TinkerHap uniquely merges external phasing blocks into its read-based framework while maintaining block continuity, which differs from existing tools that do not integrate merging within a read-aware propagation step.
2. Reference bias claim
   The introduction states that read-based phasing is "independent of reference bias." While this approach is generally less susceptible to reference bias than statistical phasing, bias can still arise during the read alignment stage, potentially affecting downstream phasing. This point should be clarified.
   Great point, the differentiation between reference bias in multiple phased genomes and reference bias of the reference genome was not clear in our manuscript.- The manuscript has been revised to explain that the method itself is free from statistical bias, however mapping bias from reference-based alignment remains a known limitation
3. GIAB datasets
   The abstract mentions only the GIAB Ashkenazi trio, but later the Chinese trio is included in the analysis as well. Please clarify whether results are averaged across the two datasets.
   Thank you for noticing this. The manuscript now states that results are averaged across the Ashkenazi and Chinese GIAB trios.
4. Tool version citation
   Please clarify in the text that the comparison was made using SHAPEIT5, not an earlier version.
   Thank you for noticing this. The manuscript has been revised to clearly state that all benchmarking was performed using ShapeIT5, and the citation now matches this version.

Recommendation: Minor Revision
With additional clarification on generalizability and coverage sensitivity, this manuscript will make a valuable contribution to the field.
