# Supplementary material for: TinkerHap—a novel read-based phasing algorithm with integrated multimethod support for enhanced accuracy
Source: Gigascience. 2025 Oct 28;14:giaf138. doi: 10.1093/gigascience/giaf138 (PMC12723663; doi:10.1093/gigascience/giaf138)
Supplement: giaf138_Authors_Response_To_Reviewer_Comments_Original_Submission [file giaf138_authors_response_to_reviewer_comments_original_submission.pdf]

Author's Response To Reviewer Comments

(stylized response letter is attached separately)

We would like to thank you and the reviewers for the constructive and thoughtful peer review of our manuscript. We are hereby submitting the revised version of the manuscript along with the supplementary material. In addition, we have uploaded to the SFTP server the relevant files associated with the additions of TinkerHap+ShapeIT and ShapeIT, and we have also updated the TinkerHap-Supplementary repository on GitHub.
Email points:
"One important topic mentioned in the reports is the need to present an improved and fair assessment of the method, also with a wider scope, e.g. on a chromosome-/ genome-wide scale"

We agree that this is a very important point. Therefore, in the second part of the analysis we included a genome-wide evaluation of our method on samples from the GIAB project, which provides a comprehensive benchmark across the full genome. It is important to note that such analysis is relevant only when using long reads. Since short sequencing reads are typically ~150 bp long, and variants are typically 500-1000 bp apart, individual sequencing reads rarely cover more than one variant, so any read-based phasing method such as TinkerHap would not produce additional or meaningful phasing information. This is why we focused on our short-read analysis on the MHC class II region which is the most variable in the genome.
To emphasize these limitations of our method and make it clearer to the readers, we added the following paragraph to the discussion section:
"A key limitation of read-based phasing approaches, including TinkerHap, arises from the typical length of short sequencing reads (~150 bp) relative to the spacing between variants (500-1,000 bp on average). As a result, individual reads seldom span multiple variants, thereby yielding little to no additional or informative phasing data. This limitation confines the effective application of read-based phasing methods like TinkerHap primarily to long-read sequencing projects, where reads are sufficiently extended to span multiple variants, or to genomic regions exhibiting high variant density (greater than approximately 1 variant per 100 bp), such as the human leukocyte antigen (HLA) locus."
"In addition to addressing the reviewers' comments, please register any new software application in the bio.tools and SciCrunch.org databases to receive RRID (Research Resource Identification Initiative ID) and biotoolsID identifiers, and include these in your manuscript. Computational workflows should be registered in workflowhub.eu and the DOIs cited in the relevant places in the manuscript. These will facilitate tracking, reproducibility and re-use of your tool."

We thank you and the reviewers for pointing out these important databases. We have registered TinkerHap in the bio.tools and SciCrunch.org databases, and the manuscript now includes the assigned biotoolsID and RRID identifiers to facilitate tracking, reproducibility, and reuse of the tool. Regarding WorkflowHub.eu, we believe this registry is less applicable to TinkerHap, as TinkerHap is a standalone software tool for read-based variant phasing rather than a multi-step computational workflow.

Reviewer reports:
Reviewer #1.1: "The benchmarking was only performed on MHC Class II, which is a relatively small and easy to phase region based on the high level of heterozygosity. How does the statistics look when applied to the whole genome? After generating the phased read set, what % of reads can be accurately assigned to the original haplotype in the whole genome scale? To benchmark the latter, I would recommend doing it on HG002 phased variants and reads by using the HG002Q100 genome (https://github.com/marbl/hg002) - i.e. map the classified reads and calculate the coverage and accuracy based on where the reads align to. I would be curious to see how the MHC Class II phased read alignment looks like on the HG002Q100 truth assembly, on each haplotype."

Thank you very much for your thoughtful comments and suggestions.
Regarding the benchmarking on MHC Class II, we acknowledge that this region benefits from high

heterozygosity, making it particularly suitable for phasing. We agree that it is essential to test TinkerHap on a genome-wide scale. Therefore, we have already analyzed TinkerHap's performance on the whole-genome scale for HG002 and an additional GIAB sample, with the results presented in Table 2.
For short-reads, since short sequencing reads are typically ~150 bp long, and variants are typically 500-1000 bp apart, individual sequencing reads rarely cover more than one variant, so applying any read-based phasing method such as TinkerHap on short reads would not produce additional or meaningful phasing information to many regions of the genome. To address this, we have now added a caveat to the Discussion section highlighting this limitation of read-based phasing methods like TinkerHap:
"A key limitation of read-based phasing approaches, including TinkerHap, arises from the typical length of short sequencing reads (~150 bp) relative to the spacing between variants (500-1,000 bp on average). As a result, individual reads seldom span multiple variants, thereby yielding little to no additional or informative phasing data. This limitation confines the effective application of read-based phasing methods like TinkerHap primarily to long-read sequencing projects, where reads are sufficiently extended to span multiple variants, or to genomic regions exhibiting high variant density (greater than approximately 1 variant per 100 bp), such as the human leukocyte antigen (HLA) locus."

Regarding the MHC Class II phased read alignment performance on truth assembly such as HG002Q100, it is important to note that we benchmarked TinkerHap on approximately 1,000 diverse short-read samples of trios, with truth phasing learned from parent-offspring inheritance.
Reviewer #1.2: "When showing benchmarking results, key features are missing - 1) number of heterozygous variant sites are used for phasing, in addition to the Phased % (what's the denominator here?), 2) number of phase blocks, phase block NG50 and total length and 3) Show the NGx length distribution by plotting the cumulative covered genome length as a function of the longest to shortest phase block."
We agree, and added the number of heterozygous variant sites used for phasing to the manuscript as well as the number of phase blocks, phase block N50 and the total length of the phased blocks.
Reviewer #1.3: "After phasing the variants (and reads), are the authors accurately able to type the HLA Class II genes? The goal of MHC phasing is to accurately genotype the HLA-genes. It is unclear to me why the authors applied their phasing on the 1,040 parent-offspring trios. I agree that it is 'phasable', however, it is unclear what the motivation here is - the MHC Class II is particularly known to have linked HLA types (e.g., HLA-DRB3 and HLA-DRB5 are inherited together depending on the HLA-DRB1 type, while in some haplotypes HLA-DRB3 is entirely missing), and depending on the HLA types and because the reference is incompletely representing this locus, there are multiple tools developed for genotyping this locus. I would be more convinced if the authors could show the HLA genotyping accuracy together based on their phasing method."
We agree that accurate typing of HLA Class II genes is very important. In this work we chose to evaluate phasing performance based on truth datasets learned from trios, which is the golden standard for phasing evaluation. Testing the accuracy of phasing based on the accuracy of typing might introduce biases due to the different methods the reference typing sets were created by.
Reviewer #1.4: "Is it possible to use additional data types to further extend the phase blocks, by using datasets such as low coverage PacBio data in addition to the short-read WGS? How about phasing with linked-reads or Hi-C? Both Whatshap and HapCut2 are specifically designed to combine such short and long-range datasets, giving the advantage of using such tools."
We agree that long reads such as PacBio and other techniques such as Hi-C can contribute to evaluating TinkerHap performance and to extend phasing blocks. Therefore, we have analyzed TinkerHap's performance on PacBio long reads and the results are presented in Table 2. We did not have access to Hi-C linked reads in combination with its raw data sequencing reads, and we believe that such broader comparison that integrates multiple additional technologies and tools would be better suited to a larger dedicated study, and we view this as an interesting direction for future work.
Reviewer #1.5: "The authors claim their method is free from reference bias, which I strongly disagree. Using a bam file aligned to a reference inherently has the issue of mapping biases, so any such tools are limited by the reads that aligns incorrectly. Repeats, especially copy number variable region with collapses in the reference are very difficult to accurately phase. Any large structural variant not properly represented in the reference will cause problems due to unmapped reads."
Very good point, the differentiation between reference bias in multiple phased genomes and reference bias of the reference genome was not clear in our manuscript. The manuscript has been revised to explain that the method itself is free from statistical bias, however mapping bias from reference-based alignment

remains a known limitation
Reviewer #1.6: "In Methods, 2nd section - I would suggest to use allele 1 and allele 2 instead of 'reference' and 'alternative' in the equation and the code. This will increase the number of heterozygous 'phasable' variants that does not carry any reference allele."
We thank the reviewer for this excellent suggestion. The manuscript and corresponding code have been updated to use "allele 1" and "allele 2" instead of "reference" and "alternative" in the relevant section.
Reviewer #2.1: "The authors are missing experiments for long-read based phasing. How does TinkerHap performs with ShapeIT on PacBio long-reads? I would suggest the authors using the same phasing method class as their short-read analysis: TinkerHap+ShapeIT; TinkerHap; WhatsHap; HapCUT2; ShapeIT. Also I believe ShapeIT is capable to take long-read SNV/INDEL calls as vcf."
We thank the reviewer for this valuable comment and fully agree with the suggestion. In response, we have extended our PacBio long-read benchmarking to include both TinkerHap+ShapeIT and ShapeIT alone, alongside the other methods. These additions provide a more complete and balanced evaluation of the different phasing strategies, and the results are now included in Table 2 of the revised manuscript.
Reviewer #2.2: "Following up on the point 1, the experimental design of this study is quite skewed. WhatsHap is not suitable for short-read sequencing data. It does not make sense to apply WhatsHap on short-read data."
This is a very good point. We compared WhatsHap on PacBio long reads (table 2), and used it for short reads only on the MHC class II region, because this region contains sufficient variant density for effective read-based phasing even with short-reads, making it comparable in applicability to TinkerHap. We have now also added the number of heterozygous variant sites used for phasing, which allows the reader a better understanding of the framework that the different methods were compared in
Reviewer #2.3: "I would caution the authors to read and potentially compare with SAPPHIRE (https://doi.org/10.1371/journal.pgen.1011092). This is a method that developed by the ShapeIT team for incorporating long-read sequencing data and ShapeIT. "
SAPPHIRE is a great tool for phasing, that includes ancestry-aware phasing of large-scale genotype datasets. However it does not accept a .bam or .cram alignment files as input, and does not include a read-based feature. In this work we focused on benchmarking against well-established and widely adopted read-based phasing methods to provide a stable and recognized reference frame. Combining TinkerHap with additional categories of phasing tools in order to get better phasing is a great option for future work.
Reviewer #2.4: "To better justify the hybrid strategy, I recommend adding an analysis of sites where TinkerHap and ShapeIT disagree. Are these differences due to reference bias, read coverage, variant type, or true ambiguity? Such an evaluation would help users understand when to rely on the read-based output vs. ShapeIT, and enhance confidence in the merging strategy."
An in-depth disagreement analysis should be very interesting and insightful. Since in many cases the determination of the reason for discrepancy might be impossible to do with certainty and can cause bias in the analysis, the major difference between ShapeIT and TinkerHap is that ShapeIT (and other statistical phasing algorithms) is less likely to accurately phase rare variants, while TinkerHap (and other read-based algorithms) are not affected by the frequency of the variant, but rather by the density of the variants and length of reads. For example, a previous study has shown that for ShapeIT, the switch error rate (SER) increases as the minor allele frequency (MAF) decreases, particularly for MAF below 1% (Choi et al., 2018). To make it clearer to the readers, we have added to the manuscript the following paragraph in the discussion:
"An important advantage of TinkerHap, as well as all read-based algorithms, is that it is less likely to be affected by the rarity of a variant (Choi et al., 2018). However, the density of the variants in combination with the length of the sequencing read are key factors for the performance of TinkerHap. A key limitation of read-based phasing approaches, including TinkerHap, arises from the typical length of short sequencing reads (~150 bp) relative to the spacing between variants (500-1,000 bp on average). As a result, individual reads seldom span multiple variants, thereby yielding little to no additional or informative phasing data. This limitation confines the effective application of read-based phasing methods like TinkerHap primarily to long-read sequencing projects, where reads are sufficiently extended to span multiple variants, or to genomic regions exhibiting high variant density (greater than approximately 1 variant per 100 bp), such as the human leukocyte antigen (HLA) locus."

Reviewer #2.5: "I could see the versions of the software in the supplementary github, but I think it is also important to include those in the manuscript. For example, shapeIT 2-5 are having quite different functions. The citation for ShapeIT in the manuscript is for ShapeIT 2, but the program that has been used

is for ShapeIT 5. "
Thank you for noticing this. The manuscript has been revised so that the citation now correctly matches the version of ShapeIT that was used.
Reviewer #2.6: "Need to mention the benchmarking hardware information for runtime comparison."
Thank you for pointing this out. The benchmarking hardware information was already included in the supplementary material, but we now added it also to the main manuscript.
Reviewer #2.7: ""...a novel and unique phasing algorithm..." -> "...a novel phasing algorithm..."
Thank you for ponting this out - The wording has been updated accordingly in the manuscript.
Reviewer #3.1: "Limited scope of benchmarking
The evaluation on the highly polymorphic MHC class II region is appropriate for highlighting TinkerHap's strengths in phasing rare variants in variable regions. However, the current evaluation on short -read based phasing is based on a ~700 kb region selected for its high variant density, which limits the generalizability of the findings. Since the manuscript emphasizes improved performance in regions with sparse genetic variation, it would strengthen the work to include chromosome-wide or genome-wide benchmarks, particularly on short-read data. This would also provide a more balanced comparison with tools like SHAPEIT5, which predictably underperform in the MHC class II region due to their reliance on population allele frequencies and linkage disequilibrium patterns that are less effective for rare or private variants."
Thank you very much for your thoughtful comments and suggestions.
Regarding the benchmarking on MHC Class II, we acknowledge that this region benefits from high heterozygosity, making it particularly suitable for phasing. We agree that it is essential to test TinkerHap on a genome-wide scale. Therefore, we have already analyzed TinkerHap's performance on the whole-genome scale on GIAB samples, with the results presented in Table 2.
For short-reads, since short sequencing reads are typically ~150 bp long, and variants are typically 500-1000 bp apart on average in the genome, individual sequencing reads rarely cover more than one variant, so applying any read-based phasing method such as TinkerHap on short reads would not produce additional or meaningful phasing information to most regions of the genome. To address this, we have now added a caveat to the Discussion section highlighting this limitation of read-based phasing methods like TinkerHap:
"A key limitation of read-based phasing approaches, including TinkerHap, arises from the typical length of short sequencing reads (~150 bp) relative to the spacing between variants (500-1,000 bp on average). As a result, individual reads seldom span multiple variants, thereby yielding little to no additional or informative phasing data. This limitation confines the effective application of read-based phasing methods like TinkerHap primarily to long-read sequencing projects, where reads are sufficiently extended to span multiple variants, or to genomic regions exhibiting high variant density (greater than approximately 1 variant per 100 bp), such as the human leukocyte antigen (HLA) locus."
We have also revised the text to remove the reference to "sparse genetic variation".
Reviewer #3.2: "Coverage and scalability
The manuscript describes TinkerHap as scalable, but since the algorithm relies on overlapping reads, it is unclear how its performance varies with sequencing depth. Including a figure or supplementary analysis showing phasing accuracy, runtime, and memory usage at different coverage levels (particularly for short-read data) would help support this claim and guide users on appropriate coverage requirements."
Thank you pointing this out. The manuscript includes comparisons of runtime and memory usage, which offer practical insights into the tool's scalability relative to other methods. To prevent any overstatement, we have removed the phrase "and scalability" from the algorithm description.
Reviewer #3.3: "Clarify algorithmic novelty
It would be helpful to elaborate on how TinkerHap's read-based phasing algorithm differs from existing approaches such as the weighted Minimum Error Correction (wMEC) framework implemented in WhatsHap. For example, what specifically enables TinkerHap's read-based mode to produce longer haplotype blocks than other read-based tools?"
The key difference between TinkerHap and existing approaches such as WhatsHap lies in the algorithmic strategy used to address this NP-hard problem. WhatsHap's runtime complexity is O(2k m), where k is the maximum read coverage at any variant site, and m is the number of SNP positions. To avoid exponential runtime, WhatsHap enforces an internal coverage cap (flag: "--internal-downsampling"), with a default k=15 and a hard limit of k=23. As shown in Figure 2 of the WhatsHap paper, reducing coverage increases both the error rate and the proportion of unphasable positions.
In our experiments, we used data from the UK BioBank. This data has an average coverage of x32.5. At

this depth, WhatsHap would either (i) run with an unfeasible runtime if coverage is not capped, or (ii) require downsampling, leading to higher error rates and more unphasable positions.
In contrast, TinkerHap formulates read-based phasing as a K=2 clustering problem. It takes an iterative greedy expansion approach, with an overall polynomial runtime. Like other clustering algorithms (e.g., K-Means), TinkerHap is sensitive to initialization. However, this can be mitigated with multiple runs with different initializations.
To conclude, TinkerHap enjoys a polynomial runtime, while harnessing the full benefits of a high coverage to produce longer haplotype blocks with fewer errors and unphasable sites.
Reviewer #3.4: "Data description
A brief characterization of the input datasets, such as the sequencing depth, as well as the number and average genomic distance of heterozygous variants in the MHC class II region and the GIAB trio data would provide important context for interpreting the reported phasing accuracy and haplotype block lengths."
- We agree it is very important, and so sequencing depth and the number of heterozygous variant sites used for phasing have now been added to the manuscript.
Reviewer #3.5: "Manuscript structure
Since the algorithm itself is the core novel contribution, it should be part of the results section, as well as the description of the evaluation currently in placed in the discussion. According to GigaScience's Technical Note guidelines, the method section should be reserved for "any additional methods used in the manuscript, that are not part of the new work being described in the manuscript."
Thank you very much for your thoughtful suggestion regarding the manuscript's structure and for referencing GigaScience's Technical Note guidelines. We greatly appreciate your attention to detail in ensuring alignment with journal standards.
Upon careful review of the guidelines, we recognize that the Methods section is intended for additional methods not part of the new work, while the Findings section should detail the implementation, availability, and testing of the novel contribution in a reproducible manner. In our manuscript, we placed the TinkerHap algorithm description in Methods to provide a dedicated space for the technical details needed for reproduction, as this aligns with another requirement of the technical guidelines for the method section - "This section should provide enough detail to allow other researchers to interpret and repeat the study."
We also think that conventional expectations in bioinformatics papers are that researchers often look to Methods for algorithmic specifics to interpret and repeat the work, while focusing on the results section for evaluation and benchmarking.
To better adhere to the guidelines without major restructuring, we have added cross-references and a subsection in Results explicitly linking to the algorithm's implementation details, emphasizing reproducibility. ~Based on the algorithm described in the Methods section...~
Reviewer #3.6: "Novelty of hybrid approach
While TinkerHap's ability to integrate externally phased haplotypes is valuable, similar functionality exists in other tools, for example, SHAPEIT can accept pre-phased scaffolds (including those generated from read-based phasing), and WhatsHap supports trio-based phasing. Consider refining the language to more precisely describe what is uniquely implemented in TinkerHap's hybrid strategy. It would be interesting to see how the presented results of using SHAPEIT's phasing output as input for TinkerHap compare to an approach of feeding TinkerHap's read-based phasing results into SHAPEIT."
Great point. The introduction text now specifies that TinkerHap uniquely merges external phasing blocks into its read based framework while maintaining block continuity, which differs from existing tools that do not integrate merging within a read aware propagation step.
Reviewer #3.7: "Reference bias claim
The introduction states that read-based phasing is "independent of reference bias." While this approach is generally less susceptible to reference bias than statistical phasing, bias can still arise during the read alignment stage, potentially affecting downstream phasing. This point should be clarified."
Great point, the differentiation between reference bias in multiple phased genomes and reference bias of the reference genome was not clear in our manuscript.- The manuscript has been revised to explain that the method itself is free from statistical bias, however mapping bias from reference-based alignment remains a known limitation
Reviewer #3.8: "GIAB datasets
The abstract mentions only the GIAB Ashkenazi trio, but later the Chinese trio is included in the analysis as well. Please clarify whether results are averaged across the two datasets."
Thank you for noticing this. The manuscript now states that results are averaged across the Ashkenazi and Chinese GIAB trios.

Reviewer #3.19 " Tool version citation
Please clarify in the text that the comparison was made using SHAPEIT5, not an earlier version."
Thank you for noticing this. The manuscript has been revised to clearly state that all benchmarking was performed using ShapeIT5, and the citation now matches this version.

Cookie Preference Center

We use cookies which are necessary to make our site work. We may also use additional cookies to analyze, improve and personalize our content and your digital experience. For more information, see our Cookie Policy.

You may choose not to allow some types of cookies. However, blocking some types may impact your experience of our site and the services we are able to offer. See the different category headings below to find out more or change your settings.

Allow all

Manage Consent Preferences

Strictly Necessary Cookies

Always active

These cookies are necessary for the website to function and cannot be switched off in our systems. They are usually only set in response to actions made by you which amount to a request for services, such as setting your privacy preferences, logging in or filling in forms. You can set your browser to block or alert you about these cookies, but some parts of the site will not then work.

Functional Cookies

These cookies enable the website to provide enhanced functionality and personalisation. They may be set by us or by third party providers whose services we have added to our pages. If you do not allow these cookies then some or all of these services may not function properly.

Performance Cookies

These cookies allow us to count visits and traffic sources so we can measure and improve the performance of our site. They help us to know which pages are the most and least popular and see how visitors move around the site.

Targeting Cookies

These cookies may be set through our site by our advertising partners. They may be used by those companies to build a profile of your interests and show you relevant adverts on other sites. If you do not allow these cookies, you will experience less targeted advertising.

Cookie List

Clear

Apply Cancel

Consent Leg.Interest

Confirm my choices

Powered by onetrust
