# Supplementary material for: TinkerHap—a novel read-based phasing algorithm with integrated multimethod support for enhanced accuracy
Source: Gigascience. 2025 Oct 28;14:giaf138. doi: 10.1093/gigascience/giaf138 (PMC12723663; doi:10.1093/gigascience/giaf138)
Supplement: giaf138_Reviewer_1_Report_Original_Submission — Arang Rhie -- 6/9/2025 [file giaf138_reviewer_1_report_original_submission.pdf]

Reviewer Report

Title: TinkerHap - A Novel Read-Based Phasing Algorithm with Integrated Multi-Method Support for Enhanced Accuracy

Version: Original Submission **Date:** 6/9/2025

Reviewer name: Arang Rhie

Reviewer Comments to Author:

The authors present TinkerHap, a tool that accepts a variant call set and read alignment, and assigns heterozygous variants and reads to a particular haplotype based on a greedy pairwise distance-based classification. It accepts a pre-phased VCF as an option to further extend phased blocks.The results sound neat with statistics making it look the greatest compared to current state-of-the-art read alignment based phasing methods such as HapCut2, WhatsHap, and ShapeIT which uses statistical inference from reference panel data. However, there are several aspects the authors need to address to make their results more compelling.1. The benchmarking was only performed on MHC Class II, which is a relatively small and easy to phase region based on the high level of heterozygosity. How does the statistics look when applied to the whole genome? After generating the phased read set, what % of reads can be accurately assigned to the original haplotype in the whole genome scale? To benchmark the latter, I would recommend doing it on HG002 phased variants and reads by using the HG002Q100 genome (https://github.com/marbl/hg002) - i.e. map the classified reads and calculate the coverage and accuracy based on where the reads align to. I would be curious to see how the MHC Class II phased read alignment looks like on the HG002Q100 truth assembly, on each haplotype.2. When showing benchmarking results, key features are missing - 1) number of heterozygous variant sites are used for phasing, in addition to the Phased % (what's the denominator here?), 2) number of phase blocks, phase block NG50 and total length and 3) Show the NGx length distribution by plotting the cumulative covered genome length as a function of the longest to shortest phase block.3. After phasing the variants (and reads), are the authors accurately able to type the HLA Class II genes? The goal of MHC phasing is to accurately genotype the HLA-genes. It is unclear to me why the authors applied their phasing on the 1,040 parent-offspring trios. I agree that it is 'phasable', however, it is unclear what the motivation here is - the MHC Class II is particularly known to have linked HLA types (e.g., HLA-DRB3 and HLA-DRB5 are inherited together depending on the HLA-DRB1 type, while in some haplotypes HLA-DRB3 is entirely missing), and depending on the HLA types and because the reference is incompletely representing this locus, there are multiple tools developed for genotyping this locus. I would be more convinced if the authors could show the HLA genotyping accuracy together based on their phasing method.4. Is it possible to use additional data types to further extend the phase blocks, by using datasets such as low coverage PacBio data in addition to the short-read WGS? How about phasing with linked-reads or Hi-C? Both Whatshap and HapCut2 are specifically designed to combine such short and long-range datasets, giving the advantage of using such tools.5. The authors claim their method is free from reference bias, which I strongly disagree. Using a bam file aligned to a reference inherently has the issue of mapping biases, so any such tools are limited by the reads that aligns incorrectly. Repeats, especially copy

number variable region with collapses in the reference are very difficult to accurately phase. Any large structural variant not properly represented in the reference will cause problems due to unmapped reads.6. In Methods, 2nd section - I would suggest to use allele 1 and allele 2 instead of 'reference' and 'alternative' in the equation and the code. This will increase the number of heterozygous 'phasable' variants that does not carry any reference allele.

Level of Interest

Please indicate how interesting you found the manuscript: Choose an item.

Quality of Written English

Please indicate the quality of language in the manuscript: Choose an item.

Declaration of Competing Interests

Please complete a declaration of competing interests, considering the following questions:

- Have you in the past five years received reimbursements, fees, funding, or salary from an organisation that may in any way gain or lose financially from the publication of this manuscript, either now or in the future?
- Do you hold any stocks or shares in an organisation that may in any way gain or lose financially from the publication of this manuscript, either now or in the future?
- Do you hold or are you currently applying for any patents relating to the content of the manuscript?
- Have you received reimbursements, fees, funding, or salary from an organization that holds or has applied for patents relating to the content of the manuscript?
- Do you have any other financial competing interests?
- Do you have any non-financial competing interests in relation to this paper?

If you can answer no to all of the above, write 'I declare that I have no competing interests' below. If your reply is yes to any, please give details below.

I declare that I have no competing interests.

I agree to the open peer review policy of the journal. I understand that my name will be included on my report to the authors and, if the manuscript is accepted for publication, my named report including any attachments I upload will be posted on the website along with the authors' responses. I agree for my report to be made available under an Open Access Creative Commons CC-BY license (http://creativecommons.org/licenses/by/4.0/). I understand that any comments which I do not wish to be included in my named report can be included as confidential comments to the editors, which will not be published.

Choose an item.

To further support our reviewers, we have joined with Publons, where you can gain additional credit to further highlight your hard work (see: https://publons.com/journal/530/gigascience). On publication of this paper, your review will be automatically added to Publons, you can then choose whether or not to claim your Publons credit. I understand this statement.

Yes Choose an item.
