# Supplementary material for: TinkerHap—a novel read-based phasing algorithm with integrated multimethod support for enhanced accuracy
Source: Gigascience. 2025 Oct 28;14:giaf138. doi: 10.1093/gigascience/giaf138 (PMC12723663; doi:10.1093/gigascience/giaf138)
Supplement: giaf138_Reviewer_2_Report_Original_Submission — Yilei Fu, Ph.D. -- 6/15/2025 [file giaf138_reviewer_2_report_original_submission.pdf]

Reviewer Report

Title: TinkerHap - A Novel Read-Based Phasing Algorithm with Integrated Multi-Method Support for Enhanced Accuracy

Version: Original Submission **Date:** 6/15/2025

Reviewer name: Yilei Fu, Ph.D.

Reviewer Comments to Author:

TinkerHap is a read-based phasing algorithm designed to accurately assign alleles to parental haplotypes using sequencing reads.General comments:1. The manuscript would greatly benefit from the inclusion of a flowchart or schematic overview of the TinkerHap algorithm. Given that the method incorporates multiple componentsâ€"including read-based phasing, pairwise distance-based unsupervised classification, and optional integration with statistical phasing tools like ShapeITâ€"a visual diagram would help readers grasp the workflow more intuitively.Major comments:1. The authors are missing experiments for long-read based phasing. How does TinkerHap performs with ShapeIT on PacBio long-reads? I would suggest the authors using the same phasing method class as their short-read analysis: TinkerHap+ShapeIT; TinkerHap; WhatsHap; HapCUT2; ShapeIT. Also I believe ShapeIT is capable to take long-read SNV/INDEL calls as vcf.2. Following up on the point 1, the experimental design of this study is quite skewed. WhatsHap is not suitable for short-read sequencing data. It does not make sense to apply WhatsHap on short-read data.3. I would caution the authors to read and potentially compare with SAPPHIRE (https://doi.org/10.1371/journal.pgen.1011092). This is a method that developed by the ShapeIT team for incorporating long-read sequencing data and ShapeIT.4. To better justify the hybrid strategy, I recommend adding an analysis of sites where TinkerHap and ShapeIT disagree. Are these differences due to reference bias, read coverage, variant type, or true ambiguity? Such an evaluation would help users understand when to rely on the read-based output vs. ShapeIT, and enhance confidence in the merging strategy.Minor comments:1. I could see the versions of the software in the supplementary github, but I think it is also important to include those in the manuscript. For example, shapeIT 2-5 are having quite different functions. The citation for ShapeIT in the manuscript is for ShapeIT 2, but the program that has been used is for ShapeIT 5.2. Need to mention the benchmarking hardware information for runtime comparison.3. "...a novel and unique phasing algorithm..." -> "...a novel phasing algorithm..."

Level of Interest

Please indicate how interesting you found the manuscript: Choose an item.

Quality of Written English

Please indicate the quality of language in the manuscript: Choose an item.

Declaration of Competing Interests

Please complete a declaration of competing interests, considering the following questions:

- Have you in the past five years received reimbursements, fees, funding, or salary from an organisation that may in any way gain or lose financially from the publication of this manuscript, either now or in the future?
- Do you hold any stocks or shares in an organisation that may in any way gain or lose financially from the publication of this manuscript, either now or in the future?
- Do you hold or are you currently applying for any patents relating to the content of the manuscript?
- Have you received reimbursements, fees, funding, or salary from an organization that holds or has applied for patents relating to the content of the manuscript?
- Do you have any other financial competing interests?
- Do you have any non-financial competing interests in relation to this paper?

If you can answer no to all of the above, write 'I declare that I have no competing interests' below. If your reply is yes to any, please give details below.

I declare that I have no competing interests

I agree to the open peer review policy of the journal. I understand that my name will be included on my report to the authors and, if the manuscript is accepted for publication, my named report including any attachments I upload will be posted on the website along with the authors' responses. I agree for my report to be made available under an Open Access Creative Commons CC-BY license (http://creativecommons.org/licenses/by/4.0/). I understand that any comments which I do not wish to be included in my named report can be included as confidential comments to the editors, which will not be published.

Choose an item.

To further support our reviewers, we have joined with Publons, where you can gain additional credit to further highlight your hard work (see: https://publons.com/journal/530/gigascience). On publication of this paper, your review will be automatically added to Publons, you can then choose whether or not to claim your Publons credit. I understand this statement.

Yes Choose an item.
