# Supplementary material for: TinkerHap—a novel read-based phasing algorithm with integrated multimethod support for enhanced accuracy
Source: Gigascience. 2025 Oct 28;14:giaf138. doi: 10.1093/gigascience/giaf138 (PMC12723663; doi:10.1093/gigascience/giaf138)
Supplement: giaf138_Reviewer_3_Report_Original_Submission — Julia Markowski -- 6/25/2025 [file giaf138_reviewer_3_report_original_submission.pdf]

Reviewer Report

Title: TinkerHap - A Novel Read-Based Phasing Algorithm with Integrated Multi-Method Support for Enhanced Accuracy

Version: Original Submission **Date:** 6/25/2025

Reviewer name: Julia Markowski

Reviewer Comments to Author:

In the presented Technical Note "TinkerHap - A Novel Read-Based Phasing Algorithm with Integrated Multi-Method Support for Enhanced Accuracy" by Hartmann et al., the authors introduce TinkerHap, a new hybrid phasing tool that primarily relies on read-based phasing for both short- and long-read sequencing data, but can additionally incorporate externally phased haplotypes, enabling it to build upon phase information derived from existing statistical or pedigree-based phasing approaches. This hybrid approach addresses an important and timely challenge in the field: integrating the complementary strengths of different phasing strategies to improve the accuracy and span of haplotype blocks, particularly for rare variants, or in variant-sparse genomic regions. The authors clearly articulate the limitations of existing approaches and present their solution in a manner that is both elegant and accessible. Design features such as multiple output formats and compatibility with third-party tools demonstrate a practical awareness of user needs. The authors evaluate TinkerHap using both short-read and long-read state-of-the-art benchmarking datasets, and compare its performance against commonly used phasing tools, demonstrating improvements in both phasing accuracy and haplotype block lengths. Overall, this is a well-conceived and thoughtfully implemented contribution to the phasing community.While the manuscript is overall well written, there are a few areas where additional clarification or extension would improve its impact. I recommend the following revisions to help clarify key aspects of the method, enhance the generalizability of the evaluation, and align the manuscript more closely with journal guidelines.Major Comments* (1) Limited scope of benchmarkingThe evaluation on the highly polymorphic MHC class II region is appropriate for highlighting TinkerHap's strengths in phasing rare variants in variable regions. However, the current evaluation on short -read based phasing is based on a âˆ¼700 kb region selected for its high variant density, which limits the generalizability of the findings. Since the manuscript emphasizes improved performance in regions with sparse genetic variation, it would strengthen the work to include chromosome-wide or genome-wide benchmarks, particularly on short-read data. This would also provide a more balanced comparison with tools like SHAPEIT5, which predictably underperform in the MHC class II region due to their reliance on population allele frequencies and linkage disequilibrium patterns that are less effective for rare or private variants.* (2) Coverage and scalabilityThe manuscript describes TinkerHap as scalable, but since the algorithm relies on overlapping reads, it is unclear how its performance varies with sequencing depth. Including a figure or supplementary analysis showing phasing accuracy, runtime, and memory usage at different coverage levels (particularly for short-read data) would help support this claim and guide users on appropriate coverage requirements.* (3) Clarify algorithmic noveltyIt would be helpful to elaborate on how TinkerHap's read-based phasing algorithm differs from existing approaches such as

the weighted Minimum Error Correction (wMEC) framework implemented in WhatsHap. For example, what specifically enables TinkerHap's read-based mode to produce longer haplotype blocks than other read-based tools?* (4) Data descriptionA brief characterization of the input datasets, such as the sequencing depth, as well as the number and average genomic distance of heterozygous variants in the MHC class II region and the GIAB trio data would provide important context for interpreting the reported phasing accuracy and haplotype block lengths.* (5) Manuscript structureSince the algorithm itself is the core novel contribution, it should be part of the results section, as well as the description of the evaluation currently in placed in the discussion. According to GigaScience's Technical Note guidelines, the method section should be reserved for "any additional methods used in the manuscript, that are not part of the new work being described in the manuscript."Minor Comments* (a) Novelty of hybrid approachWhile TinkerHap's ability to integrate externally phased haplotypes is valuable, similar functionality exists in other tools, for example, SHAPEIT can accept pre-phased scaffolds (including those generated from read-based phasing), and WhatsHap supports trio-based phasing. Consider refining the language to more precisely describe what is uniquely implemented in TinkerHap's hybrid strategy. It would be interesting to see how the presented results of using SHAPEIT's phasing output as input for TinkerHap compare to an approach of feeding TinkerHap's read-based phasing results into SHAPEIT.* (b) Reference bias claimThe introduction states that read-based phasing is "independent of reference bias." While this approach is generally less susceptible to reference bias than statistical phasing, bias can still arise during the read alignment stage, potentially affecting downstream phasing. This point should be clarified.* (c) GIAB datasetsThe abstract mentions only the GIAB Ashkenazi trio, but later the Chinese trio is included in the analysis as well. Please clarify whether results are averaged across the two datasets.* (d) Tool version citationPlease clarify in the text that the comparison was made using SHAPEIT5, not an earlier version.Recommendation: Minor RevisionWith additional clarification on generalizability and coverage sensitivity, this manuscript will make a valuable contribution to the field.

Level of Interest

Please indicate how interesting you found the manuscript: Choose an item.

Quality of Written English

Please indicate the quality of language in the manuscript: Choose an item.

Declaration of Competing Interests

Please complete a declaration of competing interests, considering the following questions:

- Have you in the past five years received reimbursements, fees, funding, or salary from an organisation that may in any way gain or lose financially from the publication of this manuscript, either now or in the future?
- Do you hold any stocks or shares in an organisation that may in any way gain or lose financially from the publication of this manuscript, either now or in the future?

- Do you hold or are you currently applying for any patents relating to the content of the manuscript?
- Have you received reimbursements, fees, funding, or salary from an organization that holds or has applied for patents relating to the content of the manuscript?
- Do you have any other financial competing interests?
- Do you have any non-financial competing interests in relation to this paper?

If you can answer no to all of the above, write 'I declare that I have no competing interests' below. If your reply is yes to any, please give details below.

I declare that I have no competing interests.

I agree to the open peer review policy of the journal. I understand that my name will be included on my report to the authors and, if the manuscript is accepted for publication, my named report including any attachments I upload will be posted on the website along with the authors' responses. I agree for my report to be made available under an Open Access Creative Commons CC-BY license (http://creativecommons.org/licenses/by/4.0/). I understand that any comments which I do not wish to be included in my named report can be included as confidential comments to the editors, which will not be published.

Choose an item.

To further support our reviewers, we have joined with Publons, where you can gain additional credit to further highlight your hard work (see: https://publons.com/journal/530/gigascience). On publication of this paper, your review will be automatically added to Publons, you can then choose whether or not to claim your Publons credit. I understand this statement.

Yes Choose an item.
